# Anabolic Effects of Salbutamol Are Lost Upon Immobilization

**DOI:** 10.1002/jcsm.70114

**Published:** 2025-11-06

**Authors:** Jelle C. B. C. de Jong, Tom S. O. Jameson, Rob C. Andrews, Mandy V. Dunlop, Doaa R. Abdelrahman, Andrew J. Murton, Martien P. M. Caspers, Nicole Worms, Anita van Nieuwkoop, Nanda Keijzer, Qihan Cheng, Bruno Guigas, Esther van Duijn, Wouter H. J. Vaes, Arie G. Nieuwenhuizen, Jaap Keijer, Benjamin T. Wall, Lars Verschuren, Francis B. Stephens, Anita M. van den Hoek, Marlou L. Dirks

**Affiliations:** ^1^ Department of Metabolic Health Research The Netherlands Organization for Applied Scientific Research (TNO) Leiden The Netherlands; ^2^ Human and Animal Physiology Wageningen University Wageningen The Netherlands; ^3^ Department of Microbiology and Systems Biology The Netherlands Organization for Applied Scientific Research (TNO) Leiden The Netherlands; ^4^ Department of Public Health and Sport Sciences, Faculty of Health and Life Sciences University of Exeter Exeter UK; ^5^ National Institute for Health and Care Research (NIHR) Exeter Biomedical Research Centre (BRC) Exeter UK; ^6^ Department of Surgery University of Texas Medical Branch Galveston Texas USA; ^7^ Sealy Center on Aging University of Texas Medical Branch Galveston Texas USA; ^8^ Leiden University Center for Infectious Diseases Leiden University Medical Center (LUMC) Leiden the Netherlands

**Keywords:** β2‐adrenoreceptor, anabolic resistance, cAMP, insulin resistance, muscle disuse atrophy, sarcopenia, Type 2 diabetes

## Abstract

**Background:**

Periods of muscle disuse occur during hospitalization, illness or the recovery from (sports) injury and lead to a rapid loss of muscle mass and the development of insulin resistance. Salbutamol is a fast‐acting β2‐adrenoreceptor agonist that may improve muscle protein synthesis and insulin sensitivity during experimental muscle disuse and thereby attenuate or preserve muscle mass; however, this has not yet been tested as a standalone intervention.

**Methods:**

Effects of salbutamol treatment on muscle metabolism were studied in a randomized controlled trial using a human forearm immobilization model (*n* = 20). Before and after immobilization for 2 days, we measured whole‐body glucose disposal, forearm glucose uptake and amino acid kinetics during fasting and hyperinsulinaemic–hyperaminoacidaemic–euglycemic clamp conditions using forearm balance and L‐[ring‐^2^H_5_]‐phenylalanine infusion. Underlying mechanistic effects were studied as well using a complementary murine hindleg immobilization model (2 weeks) using tracer approaches (i.e., deuterated water and ^14^C‐labelled phenylalanine) and molecular analyses (e.g., RNA‐seq and western blot).

**Results:**

In humans, salbutamol enhanced insulin‐stimulated glucose disposal on the whole‐body level (+21%, *p* = 0.010) but was unable to ameliorate the immobilization‐induced decrease in forearm glucose uptake. Salbutamol decreased the efflux of amino acids from the immobilized forearm, indicating increased muscle protein synthesis and/or inhibition of breakdown. However, this did not affect the immobilization‐induced impairment of amino acid net balance in both postabsorptive (−250%) and clamp conditions (−261%, both *p* = 0.031). In agreement, in mice, salbutamol increased cumulative muscle protein synthesis (+0.87%, *p* < 0.001) but did not result in a net gain of muscle mass upon immobilization due to an accompanying increase in muscle protein turnover (+13%, *p* < 0.001). Molecular analyses revealed immobilization inhibited salbutamol's effects on the muscle transcriptome, specifically the muscle contraction pathway (−2.1 normalized enrichment score, *p* < 0.001).

**Conclusions:**

Salbutamol increases muscle mass and glucose uptake, although these effects are limited to active but not inactive muscles. This demonstrates that the mechanism of action and efficacy of β2‐adrenoreceptor signalling are hampered upon immobilization, which offers potential for a combined treatment intervention of reintroducing muscle contraction and salbutamol administration to improve muscle mass and clinical outcomes during episodes of physical inactivity.

## 1 Introduction

Short periods of limb immobilization or bed rest, often occurring as a result of injury or illness, lead to loss of muscle mass and function. Although several mechanisms contributing to disuse‐induced muscle atrophy have been identified, including metabolic alterations such as insulin resistance (i.e., impaired insulin‐stimulated glucose uptake [1, S1, S2]) and anabolic resistance (i.e., blunted muscle protein synthetic response to protein or amino acid intake [2, S3]), the underlying mechanisms remain to be elucidated. In our recent work, we demonstrated that insulin resistance developed rapidly following the removal of muscle contraction, demonstrated by a 40% reduction in muscle glucose uptake within 2 days of forearm immobilization [3] and plateaued thereafter. This insulin resistance may not be limited to glucose metabolism but may also extend to amino acid metabolism. Anabolic resistance was clearly visible following 5 days of leg immobilization [4], with early signs present following merely 2 days of disuse [5]. This anabolic resistance was accompanied by a reduced postprandial phenylalanine net balance, which was due to an impairment in the rate of disappearance of phenylalanine in plasma, representing impaired muscle amino acid uptake following mixed meal ingestion [6]. As such, treatments to attenuate or even prevent insulin resistance and anabolic resistance are warranted to preserve muscle mass during bed rest and other conditions of disuse.

One potentially promising strategy to prevent muscle atrophy is the use of a pharmacological treatment with β2‐adrenoceptor agonist (‘β2‐agonist’) drugs [7, S4]. These drugs have been shown to affect muscle protein turnover and insulin sensitivity. Recent work demonstrated a 1 kg increase in lean mass [8] and a 27% increase in insulin sensitivity (i.e., glucose infusion rate during a hyperinsulinaemic–euglycaemic clamp [8, 9]) following 4 weeks of daily 8 inhalations (as a single dose) of the selective β2‐agonist terbutaline in healthy volunteers. The selective β2‐agonist terbutaline‐induced hypertrophy is also suggested to lead to a concomitant increase in muscle force and peak power [10]. Evidence from animal models shows that the anabolic effects of β2‐agonist administration are likely mediated via positive effects on muscle protein net balance through stimulation of muscle protein synthesis and inhibition of muscle protein breakdown [11, 12, S5], as also found in a human trial supplementing young men with salbutamol after resistance exercise [13]. For these reasons, β2‐agonists might be a potent strategy to alleviate disuse‐induced insulin resistance and anabolic resistance and thereby preserve muscle mass, yet their effect on skeletal muscle has never been investigated during a period of disuse in humans.

As such, the aim of this work was to determine the impact of β2‐agonist administration on muscle mass regulation, muscle amino acid metabolism and insulin sensitivity during immobilization. Healthy, young volunteers underwent 2 days of forearm cast immobilization, prior to and immediately following which whole‐body and forearm glucose disposal and forearm amino acid kinetics were measured. These measurements provide important insight into the uptake, release and net balance (i.e., net muscle growth) of amino acids across forearm muscles. To uncover the intramuscular mechanisms involved, forearm muscle biopsy samples are required; however, due to technical challenges with this procedure, alternative approaches are required for generating further mechanistic insight. As such, a complementary mouse study was performed using a translational mouse immobilization model for human muscle atrophy [14] to gain insight into the underlying mechanisms of the observed anabolic effects. The effects of the selective, fast‐acting β‐2 agonist salbutamol on muscle mass in both immobilized and non‐immobilized conditions were assessed to gain insight into the systemic and intramuscular processes regulated by salbutamol. We hypothesized that the administration of salbutamol during immobilization would support muscle mass maintenance by creating a more positive muscle amino acid balance and alleviating muscle insulin resistance.

## 2 Materials and Methods

### 2.1 Human Study Participants

The present study included 22 healthy young (age 23 ± 1 year) males and females, of whom 20 were included in the final analysis (characteristics in Table S1; details on dropouts under ‘Metabolic test day’). Participants attended the Royal Devon University Healthcare NHS Foundation Trust Clinical Research Facility (CRF) prior to inclusion in the study, where a routine medical screening was performed to ensure their eligibility to participate. The following exclusion criteria were applied: age under 18 or over 40 years old; BMI below 18.5 or over 30 kg m^−2^; metabolic impairment, for example, Type 1 or 2 diabetes; hypertension; chronic use of any nutritional supplements or prescribed over‐the‐counter pharmaceuticals; cardiovascular disease; a personal or family history of thrombosis, seizures, epilepsy or schizophrenia; known allergy to any of the pharmacological treatments in the study; any disorders in motor or lipid metabolism; presence of an ulcer in the stomach or gut; severe kidney problems; and pregnancy or breastfeeding. Participants were informed of the study procedures and associated risks before oral and written consent were obtained. Thereafter, body weight and height were measured, and body composition was determined by air displacement plethysmography (Bodpod; Life Measurement Inc., Concord, CA, USA). The study was part of a larger trial investigating the effects of pharmacological manipulation of substrate ability on muscle health during forearm immobilization [15]. Randomization into treatment groups was performed for the sexes separately and with block sizes of 3 via the website www.randomization.com. Blinding of the treatments and randomization of participants was performed by an investigator not involved in the study. The study was approved by the NHS Wales REC4 Research Ethics Committee (18/WA/0330) in accordance with the Declaration of Helsinki (version October 2013) and registered on clinicaltrials.gov as NCT03866512. All participants provided written informed consent prior to enrolment.

### 2.2 Experimental Outline of Human Study

Following successful screening and inclusion in the study, participants attended the CRF for a baseline metabolic test day during which the arterialized venous‐deep venous (AV‐V) forearm balance method was applied to quantify forearm nutrient balance under free‐living conditions. At least 5 days later, participants visited the CRF again for the casting visit during which a forearm cast was applied, and the blinded pharmacological treatment (i.e., 4 mg salbutamol or placebo, taken 4 times daily) and a fully controlled eucaloric diet were provided for the next 2 days. Following 2 days of forearm immobilization with pharmacological treatment and standardized nutrition, the metabolic test day was repeated, and the forearm cast removed.

### 2.3 Metabolic Test Day

Participants reported at the CRF at 8 am, after an overnight fast from 10 pm, for the metabolic test day. Although participants were in a semi‐supine position on a bed, the following three intravenous cannulas were placed: (1) anterograde in an antecubital vein of the non‐immobilized hand for intravenous infusions, (2) retrograde into a dorsal hand vein of the non‐immobilized hand for arterialized venous blood sampling (with the hand placed in a hand warmer at 55°C) and (3) retrograde into a deep‐lying antecubital vein of the (to‐be) immobilized arm to sample venous blood draining the forearm muscle bed [S6, S7]. Baseline venous blood was collected at *t* = −150 min, after which a primed (0.5 mg kg body weight^−1^), continuous (0.5 mg kg body weight^−1^ h^−1^) infusion of L‐[*ring*‐^2^H_5_]phenylalanine (CK Isotopes Ltd., Newtown Unthank, UK) was started that was infused for the entire test day. After a 2‐h pre‐infusion period, simultaneous arterialized‐venous (AV) and deep‐venous (V) blood samples were taken five times between *t* = −30 and *t* = 0 min to measure postabsorptive forearm muscle metabolism. Prior to every set of AV and V samples, brachial artery blood flow of the (to‐be) immobilized arm was determined by high‐resolution ultrasound imaging in duplex mode (~12 MHz, Apogee, 1000. SIUI, China). The luminal diameter was imaged 5 cm proximal to the antecubital fossa for a 2‐s period, and at the same anatomic location mean blood velocity was determined by integration of the pulsed‐wave Doppler signal for at least 8 cardiac cycles [S6]. Captured files were analysed semi‐automatically using Brachial Analyzer for Research, Version 6.10.2 (Medical Imaging Applications LLC, Coralville, IA, USA [S8]).

Following the collection of postabsorptive AV‐V samples, at *t* = 0 min, a hyperinsulinaemic–hyperaminoacidaemic–euglycaemic clamp was started to examine forearm muscle metabolism under insulin‐stimulated conditions. For this, the following intravenous infusions were started in the antecubital elbow vein: a primed (0–5 min: 128.2 mU m^2^ min^−1^; 5–10 min: 71.8 mU m^2^ min^−1^), continuous (from 10 min: 50 mU m^2^ min^−1^) infusion of insulin (Actrapid, Novo Nordisk Ltd., Gatwick, UK) and a primed (0.46 mL kg body weight^−1^), continuous (1.38 mL kg body weight^−1^ h^−1^) infusion of 10% Primene (Baxter, Northampton, UK), which was spiked with 7% L‐[*ring*‐^2^H_5_]phenylalanine as to minimize plasma tracer dilution. Infusion of 20% dextrose (Baxter) was started in the same cannula. Every 5 min throughout the entire 3 h clamp, a 0.5 mL blood sample was taken to measure blood glucose concentration on a bedside glucose analyser, and the amount of glucose infused was altered to maintain euglycaemia at 5.0 mmol L^−1^. Potassium chloride (0.3% KCl in 0.9% NaCl) was infused in the (to‐be) immobilized arm at a rate of 1 mL kg body weight^−1^·h^−1^ to prevent potential insulin‐ and salbutamol‐induced hypokalaemia. Data collection for the first twelve participants occurred without issues. Thereafter, unexplainable nausea and sickness occurred at the end of the clamp in five participants (of which two dropped out due to this nausea). The final four volunteers in the study received prophylactic metoclopramide hydrochloride (10 mg) intravenously at *t* = 120 min to prevent these issues. Every 30 min from the start of the clamp, brachial artery blood flow was measured, and AV and V blood samples were collected simultaneously. The final half hour of the 3 h clamp was used to collect five simultaneous AV and V blood samples, and the same steady‐state period was used to calculate the mean glucose disposal rate (GDR). The AV‐V differences in glucose and amino acid concentration multiplied by brachial artery blood flow [S9] were used to calculate forearm glucose uptake as well as amino acid balance, respectively, as published before [3]. Forearm amino acid kinetics were calculated as described in detail by ourselves elsewhere [6, 15].

### 2.4 Two Days of Forearm Immobilization

On the morning of the casting visit, participants arrived at the CRF at 8:00 to have a forearm cast applied. Which arm was cast was randomly allocated but counterbalanced for arm dominance. The first step involved applying stockinette and undercast padding to provide skin protection. Then a fibreglass (Benecast, BeneCare Medical, Manchester, UK) cast was fitted to the forearm and hand to immobilize the wrist, which resulted in a cast that extended from 5 cm distal to the antecubital fossa to 2 cm proximal to the fingertips. Participants were provided with a sling and instructions to wear that during all waking hours to keep the hand positioned above the elbow. A waterproof cover was provided to keep the cast dry while showering. Body weight was measured after casting, and this was repeated at the start of the second metabolic test day.

### 2.5 Pharmacological Intervention Human Study

During the 2 days of forearm immobilization, participants were randomly allocated to receive 4 mg salbutamol (Accord‐UK Ltd., Barnstaple, UK) or a placebo (containing microcrystalline cellulose, lactose and magnesium stearate, manufactured for this study by the Guy's and St Thomas' NHS Foundation Trust Pharmacy Manufacturing Unit) in a double‐blind manner. Pharmacological treatments were prepared by the Royal Devon University Healthcare NHS Foundation Trust Clinical Trials Pharmacy and dispensed in opaque containers by a CRF research nurse blinded to treatment. The salbutamol dose in the human study was based on previous work demonstrating a 1.6 kg increase in lean mass in a patient population using 16 mg daily for 1 year [16], as well as the use of this dosing regimen in clinical practice. Participants were instructed to orally ingest treatments four times daily, that is, at 8:00, 13:00, 18:00 and 23:00, with the last dose on the second day taken at 22:00. Compliance was monitored via hardcopy treatment logs, returned drug containers and regular communication with the participants throughout the immobilization period.

### 2.6 Dietary Intake

Participants were instructed to record their habitual food intake for three consecutive days, including two weekdays and one weekend day, prior to the immobilization period. From these food diaries, habitual energy and macronutrient intake were calculated using the online licensed Nutritics software (http://www.nutritics.com, accessed 2022). During the 48 h of forearm immobilization, participants were provided with a fully controlled eucaloric diet as used previously [3]. Participants received all meals and snacks and were instructed to eat all the food provided and nothing else. Water and non‐caloric drinks were allowed ad libitum. Energy requirements were calculated for every participant individually using basal metabolic rate (BMR; Henry equations [S10]) multiplied by an activity factor (International Physical Activity Questionnaire, IPAQ [S11]). The diet was designed to provide 1.2 g protein·kg body weight^−1^ d^−1^, with a target macronutrient composition of 50–55 energy percent (en%) carbohydrate, 30–35 en% fat, 10–15 en% protein and 2 en% dietary fibre. Compliance with the provided diet was assessed via completed food diaries, returned food containers and daily communication with the participants throughout the 48 h of immobilization.

### 2.7 Experimental Outline of Mouse Study

**FIGURE 1:**
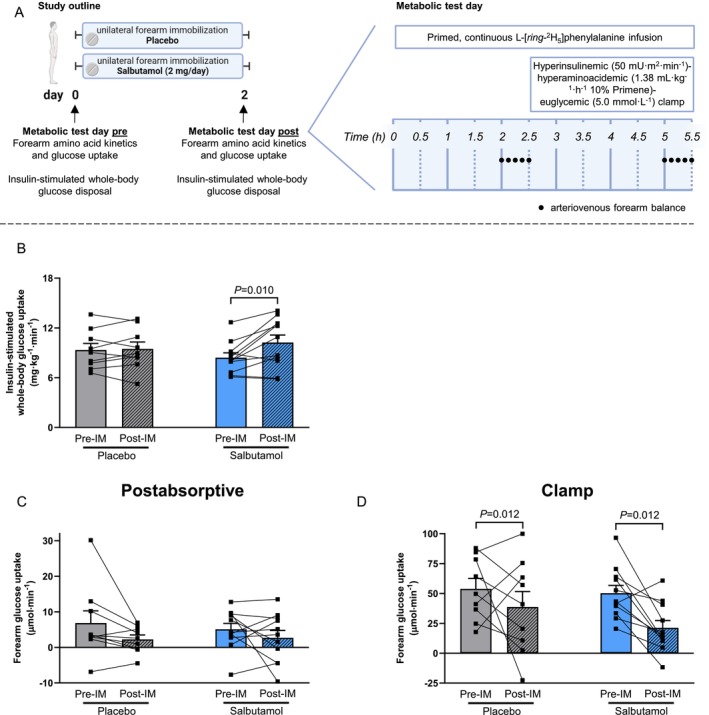
Salbutamol increased whole‐body glucose uptake, but not in the immobilized forearm muscle. (A) Schematic overview of the study design (created using Biorender.com) and execution of arteriovenous forearm balance in both postabsorptive and clamp conditions. (B) Glucose disposal rate during the final 30 min of steady state of the clamp to maintain euglycaemia at 5.0 mmol L^−1^. Immobilized forearm glucose uptake in (C) postabsorptive and (D) clamp conditions. Statistical differences were determined by three‐way ANOVA. Data of placebo (grey bars, *n* = 9) and salbutamol (blue bars, *n* = 11) groups before (open bars) and immediately after (hatched bars) immobilization are expressed as means ± SEM Data collected during both the postabsorptive state and the steady state of a hyperinsulinaemic–hyperaminoacidaemic–euglycaemic clamp are presented as indicated.

All animal care and experimental procedures were approved by the Ethical Committee on Animal Care and Experimentation (Zeist, The Netherlands; based on one ethics permit with approval reference number TNO‐534, date: 18 April 2023) and were in compliance with European Community specification regarding the use of laboratory animals. Ten‐week‐old C57BL/6J mice were obtained from Charles River Laboratories (L'Arbresle, France) and all mice were kept on a chow maintenance diet (ssniff Spezialdiäten GmbH, Soest, Germany). Male mice were chosen due to their increased translatability for human muscle atrophy [17] and because they have more muscle mass compared to female mice, making it easier to detect effects of immobilization and salbutamol treatment. The number of mice was calculated with G*Power software (Heinrich Heine University Dusseldorf, Dusseldorf, Germany) using a one‐way ANOVA with a significance threshold of 0.05, a power of 80% and an effect size f of 0.45 (as determined using pilot‐study data). Upon arrival, mice were initially housed with at least one littermate in a temperature‐controlled room on a 12‐h light–dark cycle (7:00 AM to 7:00 PM, light during daytime and dark during nighttime) and had free access to chow and water. Wood gnawing blocks and nesting material were provided. After 2 weeks, mice were housed individually for another 2 weeks and food intake was measured during the second week to assess the individual food intake. After this acclimatization period, the 2‐week intervention period started. Mice were divided into three groups which were matched for body weight and lean body mass, that is, (1) a healthy reference group receiving ad libitum food; (2) an untreated control group receiving 60% of their normal food intake, unilateral immobilization and an osmotic pump filled with vehicle (PBS); and (3) a group receiving 60% of their normal food intake, unilateral immobilization and an osmotic pump releasing 1 mg salbutamol per kg body weight per day. The dose of 1 mg salbutamol per kg body weight per day was chosen because this was observed to be effective in a previous study also employing osmotic pumps [18]. Group allocation was known by all researchers involved in the animal study. Mice in the untreated control and salbutamol‐treated groups received an osmotic pump (ALZET model 1004, Charles River Laboratories, Massachusetts, USA), which was placed underneath the skin through a small incision on the left lateral side of the mouse, just above the hip. Pump placement was performed while using isoflurane inhalation anaesthesia, and as analgesia, carprofen was added to the drinking water (0.067 mg/mL) the day prior to pump placement till 2 days after pump placement. The pump was orientated in parallel to the spine of the mouse with its opening facing the cranial side of the body. The pumps were filled with either PBS (untreated control group) or salbutamol dissolved in PBS. The pumps were incubated in PBS at 37°C for 24 h prior to inserting them in the mouse, to activate the osmotic pumps. Osmotic pumps were not removed, allowing salbutamol administration to continue until termination of the experiment, thereby capturing both acute and chronic effects. After placing the pumps, the right legs of the mice in the untreated control and salbutamol‐treated groups were immobilized (Figure 1B). This was achieved through fixation using adhesive bandage and surgical tape. The quality of the immobilization was checked daily and the bandage and tape were replaced if they became loose. The mice in the untreated control and salbutamol‐treated groups received 60% of their chow as individually assessed under ad libitum conditions, just before the dark phase, at 17:00 PM. Body weight and EchoMRI (Echo Medical Systems LTD, Houston, TX, USA) measurements were performed on Days 0, 3, 7, 10 and 13 of the intervention period, during which spot urine was also collected. Voluntary movement was measured for one consecutive night and day during the second week of the intervention period using TSE PhenoMaster V4.6.2 (TSE Systems, Bad Homburg, Germany). On Day 13, blood was drawn from the tail vein at 9:00 in the morning into EDTA‐coated tubes (Sarstedt, Nümbrecht, Germany). On Day 14, grip strength (based on all four limbs) was measured prior to termination and dissection using a grip strength meter (TSE Systems, Bad Homburg, Germany). Five trials were performed, and the trials with the highest and lowest force were excluded. The maximal force of the three remaining trials was averaged [19]. Mice were terminated on Day 14 by cervical dislocation and blood was immediately collected from the heart. Quadriceps and gastrocnemius muscles were isolated and snap‐frozen in liquid nitrogen. Soleus muscles were fixated in formalin.

### 2.8 Myofibre Type Staining

Mouse soleus muscles were cross‐sectionally cut at the thickest part of the muscle and embedded in paraffin. Cross‐sections of 5 μm were cut and attached to a slide. Slides were then deparaffinized, and the epitope was retrieved through a 20‐min incubation in 10 mM sodium citrate (pH 6) at sub‐boiling temperature using microwave heating. Free aldehyde groups were masked by a 20‐min incubation in 1.5% glycine, and blocking was performed by means of a 30‐min incubation in 5% normal goat serum. After blocking, slides were incubated with primary antibodies for MYH7 (1:200, SAB4200670, Sigma‐Aldrich, RRID:AB_3094658) and laminin (1:100. NB300‐144, Novus Biologicals, RRID:AB_10001146) diluted in 0.05% acetylated bovine serum (900.099, Aurion) and kept at 4°C overnight. Next, slides were incubated with a mixture of goat anti‐rabbit IgG Alexa Fluor 488 secondary antibody (1:500, A‐11008, Thermo Fisher, RRID:AB_143165) and goat anti‐rabbit IgG Alexa Fluor 594 secondary antibody (1:500, A‐11012, Thermo Fisher, RRID:AB_2534079). Finally, slides were counterstained using DAPI and covered using Fluoromount‐g (0100‐01, Southernbiotech). Tile scans of whole sections were made at 20× magnification using a fluorescence microscope (Leica DM6B) and a digital camera (DFC365 FX). Within each section, ImageJ [S12] was used to measure the amount of myofibres and the cross‐sectional area (CSA) of at least 35 myofibres for Type I and II myofibres separately.

### 2.9 Deuterated Water and ^14^C‐Phenylalanine Labelling

On Day 1 of the 14‐day intervention period, mice received an intraperitoneal injection of 17.5 μL D_2_O per gram body weight (84541.0500, VWR) followed by 4% D_2_O enriched drinking water until sacrifice to allow quantification of the fractional synthesis rate (FSR) of proteins [S13, S14]. Deuterium enrichment of plasma water (in blood samples) and alanine (in gastrocnemius samples) was measured by Metabolic Solutions Inc. (Nashua, NH, USA).

During the 4 weeks prior to starting the 14‐day intervention period, mice received chow enriched with 0.01 MBq ^14^C‐phenylalanine per kg (PerkinElmer, Massachusetts, USA). Once the intervention period started, the ^14^C‐phenylalanine enriched chow was replaced by normal chow. After the study, total radioactivity, ^14^C/^12^C ratios were determined in gastrocnemius and urine samples by accelerator mass spectrometry (AMS). A 1 MV multielement AMS, model 4110Bo [S15] (High Voltage Engineering, The Netherlands) was used for sample analysis. The data acquisition software used was AMS 1551. For each urine sample, 5 μL was transferred to tin foil cups and analysed for total ^14^C [S16]; the samples were dried under a stream of nitrogen. Gastrocnemius samples were freeze dried, and between 3 and 8 mg of freeze dried material was transferred to a tin foil cup. Next, samples were placed in the elemental analyser, which acted as an autosampler and combustion device for the AMS. The ^14^C/^12^C ratio, in combination with total ^12^C carbon content and the sample volume or weight, was used in further calculations to determine the total amount of ^14^C in the samples.

### 2.10 Protein Extraction and Western Blot Analysis

Snap‐frozen skeletal muscles (quadriceps) were lysed in ice‐cold buffer containing: 50 mM Hepes (pH 7.6), 50 mM NaF, 50 mM KCl, 5 mM NaPPi, 1 mM EDTA, 1 mM EGTA, 1 mM DTT, 5 mM β‐glycerophosphate, 1 mM sodium vanadate, 1% NP40 and protease inhibitors cocktail (Complete, Roche, Mijdrecht, The Netherlands) as previously reported [S17]. Western blots were performed using Ser473‐Akt, (#9271, RRID:AB_329825), Akt (#4691, RRID:AB_915783), Thr172‐AMPK (#2535, RRID:AB_331250), AMPKα (#2532, RRID:AB_330331) and LC3‐I/II (#4108) antibodies from Cell Signaling Technology. Bands were visualized by enhanced chemiluminescence and quantified using ImageJ (NIH, USA). The Ser473‐Akt/Akt, Thr172‐AMPK/AMPK and LC3‐II/LC3‐I ratios were calculated and expressed as fold change relative to healthy mice.

### 2.11 Transcriptomics Analysis

RNA extraction was performed as described previously in detail [17]. Total RNA was extracted from mouse quadriceps muscle samples using glass beads and RNA‐Bee (Campro Scientific, Veenendaal, The Netherlands). RNA integrity was examined using the RNA 6000 nano Lab‐on‐a‐Chip kit and a bioanalyser 2100 (Agilent Technologies, Amstelveen, The Netherlands). The NEBNext Ultra II Directional RNA Library Prep Kit (NEB #E7760S/L, New England Biolabs, Ipswich, MA, USA) was used to process the samples. Briefly, mRNA was isolated from total RNA using the oligo‐dT magnetic beads. After fragmentation of the mRNA, cDNA synthesis was performed, and cDNA was ligated with the sequencing adapters and amplified by PCR. The quality and yield of the amplicon were measured (Fragment Analyzer, Agilent Technologies, Amstelveen, The Netherlands). The size of the resulting product was consistent with the expected size distribution (a broad peak between 300 and 500 bp). Clustering and DNA sequencing, using the Illumina NovaSeq6000, was performed according to manufacturer's protocols of service provider GenomeScan B.V. (Leiden, The Netherlands) using a concentration of 1.1 nM of amplicon library DNA and yielding at least 15 million sequencing clusters per sample and 150 nt paired‐end reads. The genome reference and annotation file Mus_musculus. GRCm38.gencode.vM19 was used for analysis in FastA and GTF format. The reads were aligned to the reference sequence using the STAR 2.5 algorithm with default settings (https://github.com/alexdobin/STAR). Based on the mapped read locations and the gene annotation, HTSeq‐count Version 0.6.1p1 was used to count how often a read was mapped on the transcript region. These counts served as input for statistical analysis using the DEseq2 package. Subsequently, Gene Set Enrichment Analysis (GSEA) was performed based on Reactome annotated gene sets [S18]. Pathways with *p* values < 0.01 were considered to be significant.

### 2.12 Plasma and Serum Sample Analyses

Arterialized venous and deep‐venous blood samples were collected for the determination of whole‐blood glucose, plasma amino acid concentrations and [^2^H_5_]‐phenylalanine enrichments and serum insulin concentrations [15]. One part of every sample (i.e., 0.5 mL) was collected in a BD Vacutainer fluoride/oxalate tube, rolled on a tube roller for 2 min to inhibit glycolysis and subsequently analysed for whole blood glucose concentrations at bedside (YSI 2500 blood glucose analyser, Xylem Analytics UK, Tunbridge Wells, UK). A second part (i.e., 4 mL) was collected in BD Vacutainer SST II tubes, which were left to clot at room temperature for ≥ 30 min and then centrifuged at 2500 *g* at 4°C for 10 min to obtain serum samples. Arterialized serum samples were used to determine insulin concentrations (Human Insulin ELISA kit, DX‐EIA‐2935; Oxford Biosystems Ltd., Milton Park, UK). A third part of every sample (i.e., 5 mL) was collected in BD Vacutainer PST Lithium Heparin tubes and immediately centrifuged at 2500 *g* at 4°C for 10 min to obtain plasma samples. Mouse blood glucose concentrations were determined in fresh blood collected from the heart directly after the termination of the mice using a glucose hand analyser (FreeStyle Lite, Abbott, Chicago, IL, USA). Mouse plasma insulin (#90080, Crystal Chem) and IGF‐1 concentrations (MG100, R&D Systems) were determined in plasma samples collected the day prior to sacrifice using a commercial ELISA kit.

### 2.13 Statistical Analysis

All data are expressed as means ± SEM, and all analyses were performed using IBM SPS Statistics Version 28 (IBM Corp, Armonk, NY, USA). Sample size was determined based on the expected difference between immobilization‐induced glucose uptake [3] and an expected 60% attenuation thereof by β2‐agonist administration [S19, S20] using G*Power software (power = 0.80, α = 0.05). Human participant characteristics between groups at baseline were tested using a one‐way ANOVA. Data were analysed using repeated measures ANOVAs with immobilization (pre vs. post), insulin‐stimulation (basal vs. clamp) and/or time point (during test day), where relevant, as within‐subjects factors, and treatment (salbutamol vs. placebo) as between‐subjects factor. In case of a significant interaction, additional repeated measures ANOVAs were performed, with subsequent Bonferroni post hoc tests applied where necessary to locate individual differences. For the human study, all statistical testing was performed on the full dataset as well as on a subset of participants excluding those who had received metoclopramide to test for the potential effect of the drug on study outcomes. Statistical differences between mouse groups were determined non‐parametrically using a Kruskal–Wallis test followed by a Mann–Whitney *U* test. Two‐tailed *p* values are reported, and *p* values < 0.05 were considered statistically significant.

## 3 Results

### 3.1 Salbutamol Augmented Insulin‐Stimulated Glucose Uptake at the Whole‐Body Level, but Not in Immobilized Forearm Muscle of Healthy Individuals

Participant characteristics (Table S1) and habitual dietary intake (Table S2) demonstrated no baseline differences between the two treatment groups. A schematic overview of the study design and metabolic test days is given in Figure 1A. Insulin‐stimulated whole‐body glucose disposal did not change in the placebo group upon forearm immobilization but was significantly increased by 21% post‐immobilization in the salbutamol group (from 8.4 ± 0.6 to 10.3 ± 0.9 mg·kg^−1^·min^−1^, *p* = 0.010; Figure 1B). Contrary to the whole‐body level, in the immobilized forearm specifically, glucose uptake (a direct measure for insulin sensitivity) tended to decrease after immobilization in postabsorptive conditions (*p* = 0.052) and significantly decreased in clamp conditions post‐immobilization (*p* = 0.012), with no effect of salbutamol thereupon (Figure 1C **+** D). Neither forearm immobilization nor salbutamol treatment affected fasting glucose concentrations (Figure S1A). In contrast, a statistically significant interaction in fasting insulin concentrations was found (*p* = 0.049), which was driven by opposing changes between groups. Specifically, fasting insulin concentrations in the placebo group changed from 11.0 ± 0.8 to 9.4 ± 1.0 mU L^−1^ post‐immobilization (*p* = 0.116), whereas a visual increase was observed in the salbutamol group from 9.5 ± 1.0 to 10.4 ± 1.6 mU L^−1^ (*p* = 0.276; Figure S1B) post‐immobilization. Metoclopramide infusion did not affect any of the observed results.

### 3.2 Salbutamol Tended to Suppress Amino Acid Efflux From Immobilized Forearm Muscle, but Did Not Affect Net Balance

**FIGURE 2:**
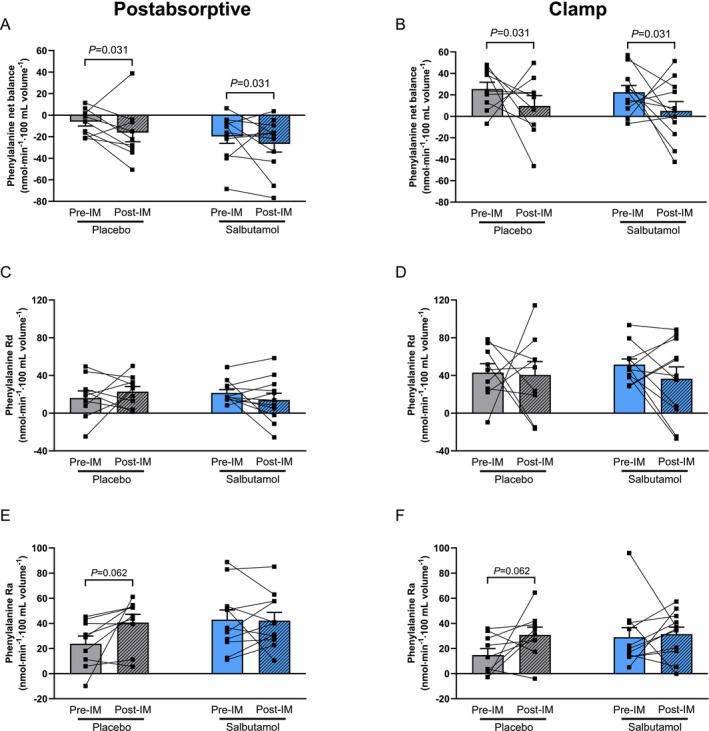
Salbutamol decreased amino acid efflux from immobilized forearm muscle but did not improve net balance. (A + B) Phenylalanine net balance before and after immobilization in healthy young males and females. (C + D) Phenylalanine rate of disappearance (Rd; i.e., measure of muscle amino acid uptake). (E + F) Phenylalanine rate of appearance (Ra; measure of muscle amino acid excretion). Statistical differences were determined by a three‐way ANOVA. Data of placebo (grey bars, *n* = 9) and salbutamol (blue bars, *n* = 11) groups before (open bars) and immediately after (hatched bars) immobilization are expressed as means ± SEM Data collected during both the postabsorptive state (left‐hand panels) and the steady state of a hyperinsulinaemic–hyperaminoacidaemic–euglycaemic clamp (right‐hand panels) are presented.

Arterialized plasma phenylalanine concentrations increased ~2‐fold from postabsorptive levels during the hyperinsulinaemic–hyperaminoacidaemic–euglycaemic clamp (from 42 ± 1 to 91 ± 2 μmol·L^−1^, *p* < 0.001; Figure S2). Specifically, this effect was driven by reduced arterialized phenylalanine concentrations post‐immobilization in the salbutamol group (*p* = 0.023; Figure S2), whereas these were unaffected in the placebo group (*p* = 0.528). A comparable response to the pharmacological treatment was observed for arterialized leucine concentrations (Figure S2). Forearm phenylalanine net balance was significantly decreased following immobilization in both postabsorptive (from −6.8 ± 4.0 to −17 ± 8.5 nmol min^−1^·100 mL^−1^) and clamp conditions (from 25.3 ± 6.4 to 9.7 ± 9.6 nmol min^−1^·100 mL^−1^, *p* = 0.031 for effect of immobilization) and was not affected by salbutamol administration (*p* = 0.942 for immobilization*treatment interaction; Figure 2A + B). The rate of disappearance of phenylalanine from plasma, a measure of its muscle uptake, was not significantly affected by immobilization or treatment in both postabsorptive and clamp conditions (Figure 2C + D). For the rate of phenylalanine appearance, a measure of its efflux from muscle, a trend for an effect of immobilization was observed (*p* = 0.092), which was driven by a trend for a ~2‐fold increase in the placebo group in both postabsorptive (from 23.8 ± 6.3 to 40.8 ± 6.5 nmol min^−1^·100 mL^−1^) and clamp conditions (from 14.8 ± 5.2 to 30.94 ± 6.2 nmol min^−1^·100 mL^−1^, *p* = 0.062), although no change was observed in the salbutamol‐treated group (from 43 ± 7.7 to 42.3 ± 6.5 nmol min^−1^·100 mL in postabsorptive conditions and from 29.1 ± 7.6 to 31.5 ± 5.6 nmol min^−1^·100 mL during clamp, *p* = 0.892; Figure 2E **+** F).

### 3.3 Salbutamol Increased Muscle Mass in Non‐Immobilized Hindlimbs Only

**FIGURE 3:**
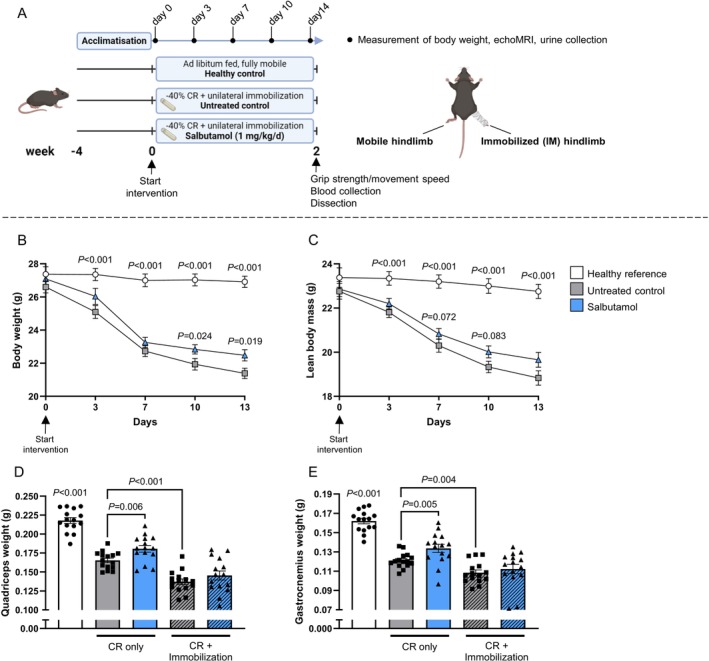
Salbutamol ameliorated muscle atrophy in muscle groups of the non‐immobilized hindlimb, but not in the immobilized hindlimb. (A) Schematic overview of the study design and mouse model, created using Biorender.com (CR = caloric restriction). (B) Body weight data and (C) lean body mass data over time during the intervention period. (D) Quadriceps and (E) gastrocnemius muscle weights as measured directly after dissection. Statistical differences were determined by a Kruskal–Wallis test followed by a Mann–Whitney *U* test. Data of healthy reference (white bars, *n* = 15), untreated control (grey bars, *n* = 15) and salbutamol (blue bars, *n* = 15) mice in both the non‐immobilized (open bars) and immobilized hindlimb (hatched bar) are presented.

A schematic overview of the study design and mouse model is given in Figure 3A. The induction of muscle atrophy via unilateral hindlimb immobilization under hypocaloric conditions induced a rapid loss of total and lean body mass, both of which were significantly decreased compared to the healthy reference group from day 3 onwards (*p* < 0.001; Figure 3B,C). Salbutamol ameliorated the loss of total body weight, which was significantly higher on Days 10 and 13 (+4% [*p* = 0.024] and +5% [*p* = 0.019] vs. untreated control, respectively; Figure 3B). Salbutamol also tended to ameliorate the loss of lean body mass on Days 7 and 10 (+3% and +4% vs. untreated control, *p* = 0.072 and *p* = 0.083, respectively; Figure 3C).

The induction of muscle atrophy resulted in a significant loss of quadriceps (−23%) and gastrocnemius muscle weights (−26%) in the non‐immobilized hindlimb (CR only, both *p* < 0.001; Figure 3D,E). Immobilization elicited an additional atrophic effect on these muscles, as demonstrated by an additional −17% (*p* < 0.001) and −10% (*p* = 0.004) weight loss for quadriceps and gastrocnemius, respectively, compared to the non‐immobilized muscles (Figure 3D,E). Interestingly, as observed in humans, salbutamol elicited a differential effect on the non‐immobilized and immobilized hindlimbs. Specifically, in the non‐immobilized hindlimb, salbutamol significantly increased muscle mass compared to untreated control mice in the quadriceps (+9%, *p* = 0.006) and gastrocnemius muscles (+10%, *p* = 0.005), whereas such a stimulatory effect of salbutamol was not observed in the immobilized hindlimb (Figure 3D,E).

### 3.4 Salbutamol Administration Was Accompanied by Increased Circulating Insulin and IGF‐1 Concentrations

**FIGURE 4:**
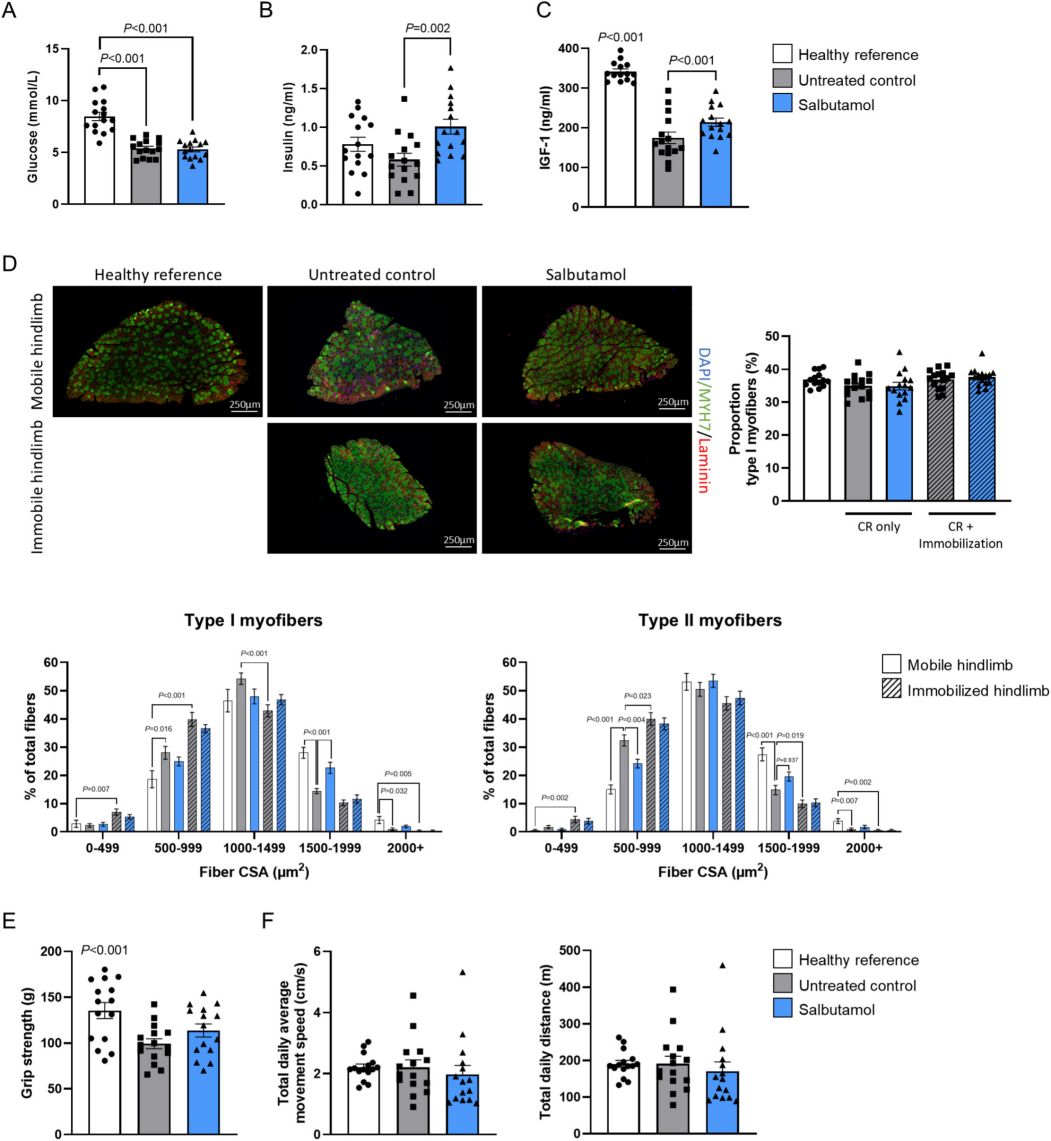
Salbutamol increased circulating insulin and IGF‐1 plasma concentrations and did not significantly affect myofibre type proportion or grip strength. (A) Blood glucose, (B) plasma insulin and (C) plasma IGF‐1 concentrations as measured in plasma collected directly after dissection. (D) Representative pictures of immunohistological staining in whole sections of soleus muscle tissue to identify Type I and II myofibre (left panel) and proportion of the different myofibre types in soleus muscle (right panel). Myofibre cross‐sectional area distribution for Type I (lower left panel) and Type II (lower right panel) myofibres. (E) Grip strength (four limbs) as measured on Day 14 prior to dissection. (F) Total daily average movement speed and distance as measured during the second week of the intervention period. Statistical differences were determined by a Kruskal–Wallis test followed by a Mann–Whitney *U* test. Data of healthy reference (white bars, *n* = 15), untreated control (grey bars, *n* = 15) and salbutamol (blue bars, *n* = 15) mice in both the non‐immobilized (open bars) and immobilized hindlimb (hatched bar) are presented (in Panel D data of *n* = 14 healthy reference mice and *n* = 13 salbutamol‐treated mice are presented).

Circulating glucose concentrations were significantly lower in the untreated control group compared to the healthy reference group (−36%, *p* < 0.001; Figure 4A), whereas insulin plasma concentrations were not different (Figure 4B). In the salbutamol group, glucose concentrations were not different compared to the untreated control group, whereas insulin plasma concentrations were significantly higher compared to the untreated control group (+73%, *p* = 0.002; Figure 4A,B). This corroborates the human data (Figure S1). Circulating IGF‐1 concentrations were significantly decreased in the untreated control group compared to the healthy reference group (−49%, *p* < 0.001), but this decline in IGF‐1 concentrations was ameliorated by salbutamol treatment (+22%, *p* < 0.001; Figure 4C). Furthermore, to study the effects of salbutamol treatment on myofibre type profile, soleus muscles were used for myofibre type staining, but differences in the proportion of myofibre type were not detected between any of the groups (Figure 4D). Distribution of myofibre CSA was determined as well for Type I and II myofibres separately (Figure 4D, lower panels). A higher proportion of both Type I and II smaller myofibres (e.g., 500–999 μm^2^) was observed in untreated control mice compared to healthy reference mice in the mobile hindlimb (*p* < 0.001). This proportion was even higher in the immobilized hindlimb of untreated control mice (*p* = 0.023). A greater proportion of larger myofibres (1500–1999 μm^2^) was present in the mobile, but not immobilized, hindlimbs of salbutamol‐treated mice compared to untreated controls (*p* < 0.001 for type I and *p* = 0.037 for type II myofibers). Grip strength at the end of the intervention period was significantly lower in the untreated control group compared to the healthy reference group (−27%, *p* < 0.001; Figure 4E). No significant effect of salbutamol on grip strength was detected. Moreover, total daily average movement speed and distance were not different between groups (Figure 4F).

### 3.5 Salbutamol Increased Muscle Protein Synthesis and Metabolic Turnover in Both Hindlimbs

**FIGURE 5:**
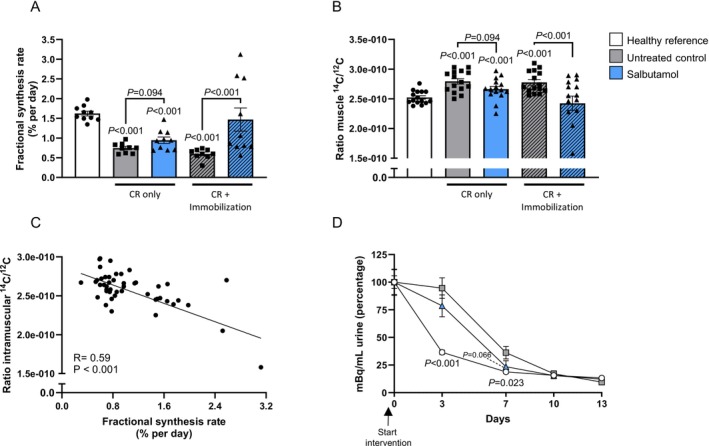
Salbutamol increased both fractional synthesis rate and the ratio of intramuscular ^14^C/^12^C in the immobilized hindlimb. (A) Fractional synthesis rate data as measured using deuterated water. (B) Ratio of intramuscular ^14^C/^12^C as a marker for the rate of turnover. (C) The correlation between fractional synthesis rate and the ratio of intramuscular ^14^C/^12^C. (D) Total radioactivity as measured in spot urine collected on Days 0, 3, 7, 10 and 13. Values on Day 0 were set to 100% for each mouse separately. Statistical differences were determined by a Kruskal‐Wallis test followed by a Mann–Whitney *U* test. Data of healthy reference (white bars, *n* = 15), untreated control (grey bars, *n* = 15) and salbutamol (blue bars, *n* = 15) mice in both the non‐immobilized (open bars) and immobilized hindlimb (hatched bar) are presented (in Panel A *n* = 10 for each group).

To investigate the contraction‐dependent effects of salbutamol on muscle mass in greater detail, muscle protein synthesis was measured by deuterated water administration. As expected, in the mouse model of muscle atrophy, the cumulative mixed muscle FSR was significantly decreased in both the non‐immobilized (−54%) and immobilized hindlimbs (−63%) in the untreated control group, compared to the healthy reference group (both *p* < 0.001, Figure 5A). Salbutamol tended to increase FSR in the muscles of the non‐immobilized hindlimb (+27%, *p* = 0.094), although a greater stimulation of muscle protein synthesis was observed in the immobilized hindlimb (+146%, *p* < 0.001; Figure 5B). The ratio ^14^C/^12^C (i.e., proxy for metabolic turnover) was significantly higher in the muscles of the untreated control group (+11% and +9% in non‐immobilized and immobilized hindlimbs respectively; both *p* < 0.001; Figure 5B) compared to the healthy group. Salbutamol decreased the muscle ^14^C/^12^C ratio in the non‐immobilized and immobilized hindlimbs (−5%, *p* = 0.094 and −13%, *p* < 0.001, respectively; Figure 5B) compared to untreated control. FSR and muscle ^14^C/^12^C ratio demonstrated a negative correlation (*R* = 0.59, *p* < 0.001; Figure 5C), indicating that higher protein synthesis rates were associated with a lower ^14^C/^12^C ratio. Total urinary radioactivity excretion was significantly higher in the untreated control group compared to the healthy reference group during the first week (*p* < 0.001 and *p* = 0.023, on Days 3 and 7, respectively; Figure 5D), demonstrating a significantly increased excretion of ^14^C‐labelled molecules in the urine of the untreated control group. Salbutamol treatment tended to decrease the total radioactive signal in the urine (*p* = 0.066, Day 7; Figure 5D), indicative of greater whole‐body ^14^C retention.

### 3.6 Salbutamol Reverted Muscle Atrophy‐Induced Changes in Muscle Transcriptome of Non‐Immobilized Muscle, an Effect Suppressed in the Immobilized Muscle

**FIGURE 6:**
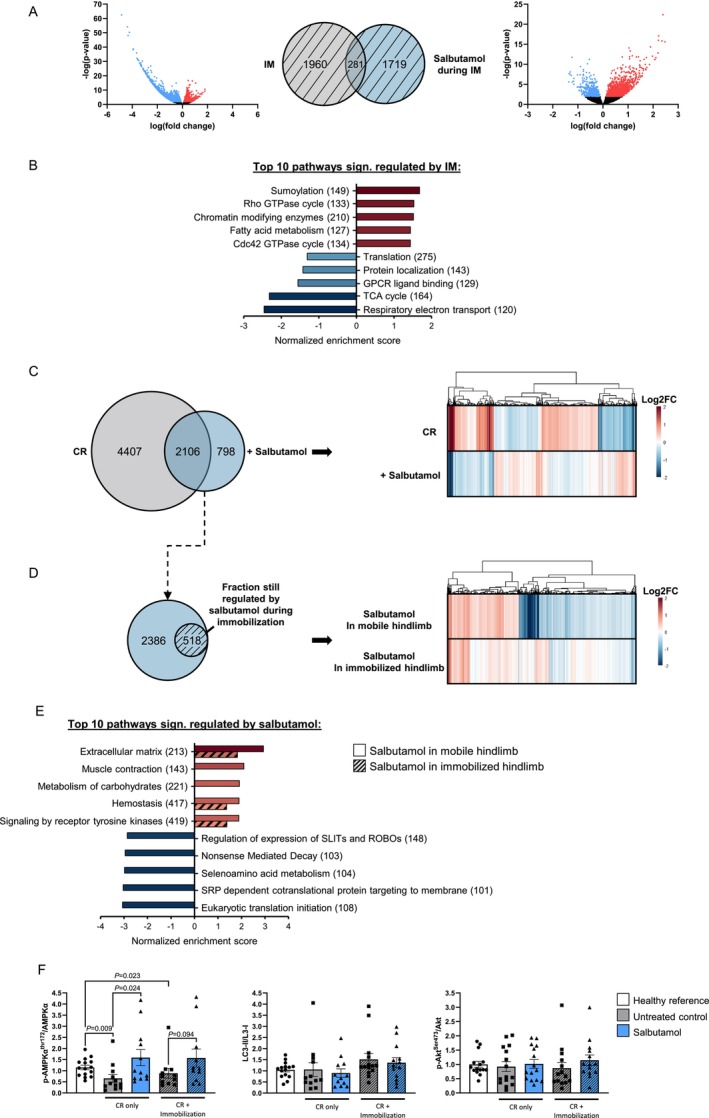
Salbutamol reversed transcriptional changes associated to muscle atrophy, but not as effectively in immobilized hindlimb. (A) Volcano plots and Venn diagram visualizing the amount of genes significantly regulated by immobilization (IM, left) and by salbutamol (right) during immobilization. (B) Top five up‐ and downregulated pathways by immobilization. Red colour indicates upregulation and blue colour indicates downregulation. Numbers behind pathway names indicate number of genes within pathways. (C) Venn diagram visualizing the amount of genes significantly regulated in the non‐immobilized hindlimb of the untreated control group (affected by caloric restriction [CR]) and by salbutamol treatment in the non‐immobilized hindlimb (left panel). Heatmap visualizing the log2FoldChange values of genes significantly regulated by caloric restriction (vs. healthy reference) or by salbutamol (vs. untreated control, leading to a total 7311 significantly regulated genes) (right panel). (D) Venn diagram visualizing the number of genes that were significantly regulated by salbutamol treatment in the non‐immobilized hindlimb and the fraction of these genes that were also significantly regulated by salbutamol in the immobilized hindlimb (left panel). Heatmap visualizing the log2FoldChange values of genes regulated by salbutamol (in total *n* = 2904) in non‐immobilized and immobilized conditions (right panel). (E) Top 10 pathways (based on normalized enrichment score) significantly regulated by salbutamol in the mobile hindpaw compared to control. Diagonally striped bars represent normalized enrichment score of the same pathway based on the comparison between the immobilized hindpaw and control. Red colour indicates upregulation and blue colour indicates downregulation. Numbers behind pathway names indicate number of genes within pathways. (F, from left to right) Ratio of phosphorylated Thr172‐AMPKα to total AMPKα, LC3‐II to LC3‐I and phosphorylated Ser473‐AKT to total AKT. Ratios were calculated and expressed as fold change relative to healthy reference mice. Statistical differences were determined by a Kruskal–Wallis test followed by a Mann–Whitney *U* test. Data of healthy reference (white bars), untreated non‐immobilized control (grey bars), untreated immobilized control (grey hatched bars), salbutamol non‐immobilized (blue bars) and salbutamol immobilized (blue hatched bars) mice (of *n* ≥ 11 per group) are presented.

To further investigate the mechanisms and pathways modulated by salbutamol and the interaction with immobilization, transcriptome analysis was performed on quadriceps muscle. Immobilization significantly regulated the expression of 2241 genes, with a small overlap in genes affected by salbutamol as well (*n* = 281; Figure 6A). Effects of immobilization and salbutamol were visualized by means of volcano plots as well (Figure 6A, left and right panel). Immobilization significantly affected pathways involved in, for example, energy metabolism and mitochondrial function (Figure 6B). In the non‐immobilized hindlimb, 6513 genes were significantly regulated due to caloric restriction, of which 2106 were significantly regulated by salbutamol as well (Figure 6C, left panel). Salbutamol mainly reverted the regulatory gene effects in the non‐immobilized hindlimb (Figure 6C, right panel).

Because salbutamol treatment ameliorated muscle atrophy in the non‐immobilized, but not the immobilized hindlimb, we investigated whether the 2904 genes altered by salbutamol were still significantly regulated in the immobilized hindlimb. Of these 2904 genes, only 518 were still significantly regulated by salbutamol in the immobilized hindlimb (Figure 6D, left panel). The log2FoldChanges revealed an inhibitory effect of immobilization on the transcriptional effects of salbutamol (Figure 6D, right panel).

The top 10 pathways significantly regulated by salbutamol in the non‐immobilized hindlimb contained significantly upregulated pathways related to the extracellular matrix, muscle contraction and carbohydrate metabolism (Figure 6E). The effect in the pathways related to muscle contraction and carbohydrate metabolism was suppressed in the immobilized hindlimb. Pathways significantly downregulated by salbutamol treatment in the non‐immobilized hindlimb contained those related to (among others) translation and the SLIT‐ROBO pathway (axon guidance). None of these pathways were affected by salbutamol in the immobilized hindlimb.

To evaluate the effects of salbutamol on key molecular regulators of muscle mass and metabolism at the protein level, we performed western blot analysis to assess the phosphorylation status of Akt (at Ser473) and AMPK (at Thr172), as well as the LC3‐II/LC3‐I ratio as a marker of autophagic flux. Salbutamol treatment significantly increased Thr172‐AMPKα phosphorylation (+243%, *p* = 0.024) in the non‐immobilized hindlimb and tended to increase AMPKα phosphorylation in the immobilized hindlimb (+178%, *p* = 0.094; Figure 6F). Both the LC3‐II/LC3‐I ratio and Ser473‐Akt phosphorylation were not different between groups (Figure 6F). Western blot membranes are included as Figure S5.

**FIGURE 7:**
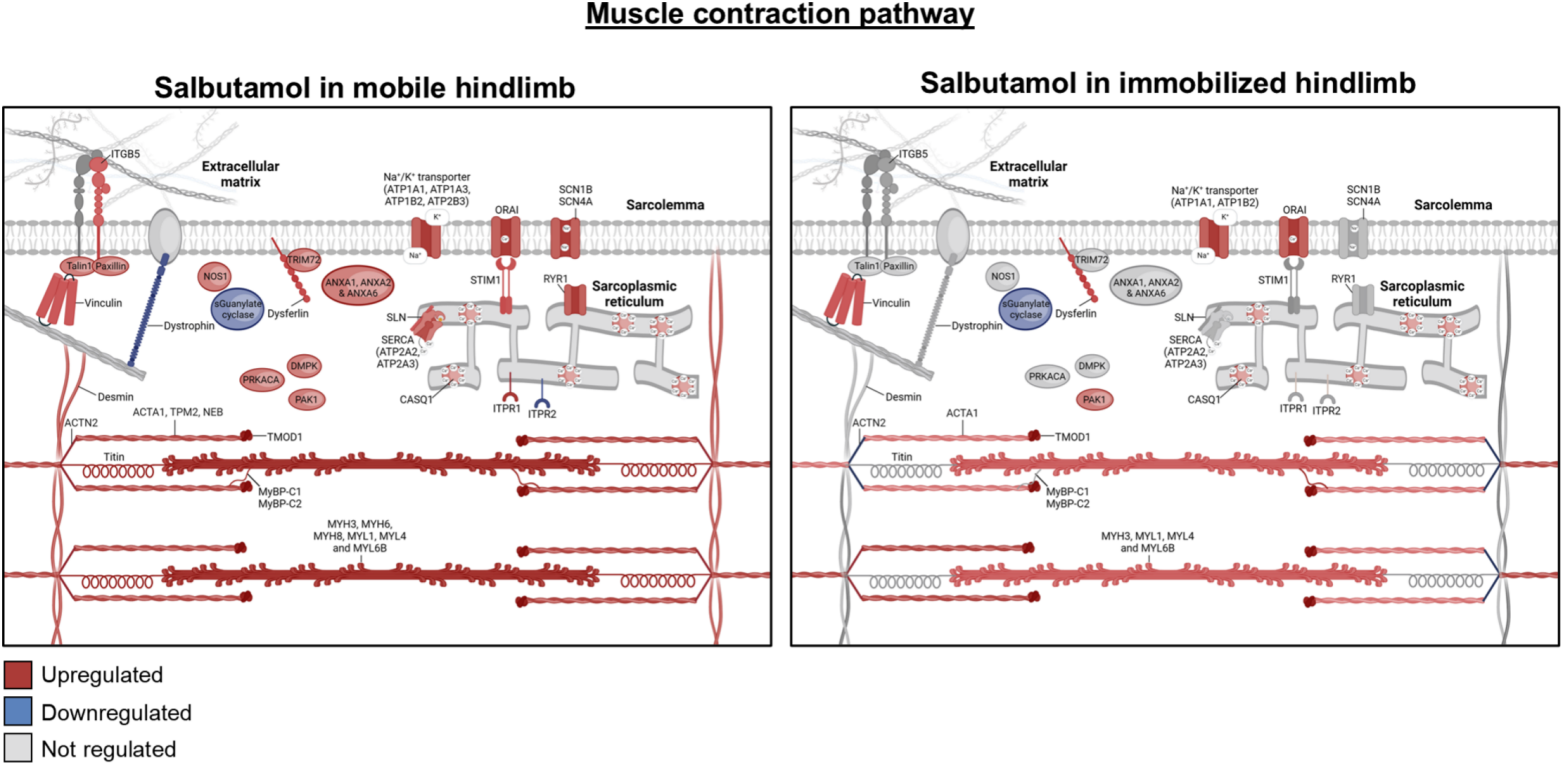
Visual representation of molecular structure and location within a myofibre of proteins encoded by genes in the muscle contraction pathway. A red colour indicates upregulation and a blue colour indicates downregulation of a gene. Data of healthy reference (*n* = 15), non‐immobilized hindlimb of untreated control (*n* = 15), immobilized hindlimb of untreated control (*n* = 15), non‐immobilized hindlimb of salbutamol treated mice (*n* = 15) and immobilized hindlimb of salbutamol treated mice (*n* = 15) were used as indicated.

An important pathway for muscle function is the muscle contraction pathway [20], which was significantly upregulated by salbutamol in the non‐immobilized (64 significantly regulated genes) but not the immobilized (23 significantly regulated genes) hindlimb. Strikingly, *PRKACA*, encoding protein kinase A (i.e., a key molecular target of salbutamol for skeletal muscle hypertrophy [13]) was significantly upregulated in the non‐immobilized hindlimb, but not in the immobilized hindlimb (Figure 7). Other genes related to sarcomeres (e.g., *TTN*, *MYH3*, *MYH6* and *MYH8*), myofibre structure (e.g., *DES*, *VCL* and *PAX*) and sarcoplasmic reticulum regulation of calcium (e.g., *RYR1*, *SERCA* subunits and *STIM1*) were found to be regulated in the non‐immobilized hindlimb, but to a much smaller degree (or not at all) in the immobilized hindlimb.

## 4 Discussion

The present study demonstrates for the first time that pharmacological treatment with the selective β2‐agonist salbutamol is unable to rescue the negative net muscle amino acid balance observed in inactive forearm muscles. Moreover, despite a significant stimulation of whole‐body glucose disposal, and in contrast to our hypothesis, salbutamol did not prevent or attenuate the development of disuse‐induced insulin resistance in the immobilized forearm muscles. Data from our muscle atrophy mouse model corroborated these findings by showing that salbutamol did not ameliorate muscle atrophy in the immobilized hindlimbs despite a stimulatory effect on muscle mass in the non‐immobilized hindlimbs. These observations reveal that muscle contraction is a prerequisite for the anabolic effect of salbutamol to occur. This was indeed confirmed by alterations in quadriceps gene expression, for example, immobilization inhibited the upregulation of the muscle contraction pathway by salbutamol. The efficacy of salbutamol to support muscle protein turnover was not completely abolished by immobilization. Specifically, we observed that salbutamol suppressed disuse‐induced postprandial forearm amino acid efflux in the human study and stimulated muscle protein synthesis rates in the mouse study, although this did not translate to a muscle mass sparing effect in either study. Altogether, our findings suggest a strongly diminished capacity of salbutamol to ameliorate the immobilization‐induced loss of skeletal muscle mass and insulin sensitivity.

Muscle disuse atrophy in humans is primarily caused by a blunted postprandial muscle protein synthesis response, in the absence of substantial changes in muscle protein breakdown [5, S21]. Because the β2‐agonist salbutamol has been shown to increase muscle protein synthesis [13], we hypothesized that administration during periods of disuse would be an effective strategy to maintain muscle mass. In our human study, immobilization tended to increase phenylalanine rate of appearance (i.e., muscle amino acid efflux rates), measured via arteriovenous forearm balance under highly controlled steady‐state clamp conditions. Importantly, this effect was fully suppressed in the volunteers who ingested salbutamol (Figure 2E,F). Unfortunately, this suggestion of muscle anabolism by salbutamol did not translate into effects on phenylalanine net balance (Figure 2A,B). Based on these findings, it can be argued that β2‐agonist administration will likely not have a substantial muscle‐sparing effect during episodes of disuse, yet due to the lack of muscle biopsy collection, the mechanistic underpinning thereof is unclear. The unilaterally immobilized mouse model confirmed the absence of effect of salbutamol on muscle mass during disuse (Figure 3D,E) and allowed us to probe the underlying mechanisms in more detail. Indeed, our mouse study revealed that salbutamol increased cumulative mixed muscle protein synthesis in immobilized conditions (Figure 5A), which is in line with previous denervation work [11]. However, due to the concomitant increase in metabolic turnover (Figure 5B), this did not result in a net gain in muscle mass (Figure 3). A significant negative correlation was found between the latter two parameters (*R* = 0.59; Figure 5C), suggesting both protein synthesis and breakdown are upregulated to a similar extent by salbutamol in immobilized conditions. This could reflect futile intracellular cycling of amino acids, failing to result in a net increase in contractile proteins due to the lack of contractile signalling. Especially because mixed muscle protein synthesis was measured, these data may reflect synthesis of non‐contractile proteins. This notion is supported by our RNA‐seq analysis, which revealed that immobilization blunted the anabolic effects of salbutamol on contractile proteins. Indeed, the majority of genes encoding contractile proteins were no longer upregulated in immobilized conditions (Figure 7). In addition, gene expression of atrogene *Fbxo32* was downregulated by salbutamol in mobile, but not in immobilized conditions. Expression of *Trim63* was increased in the mouse model, but not regulated by salbutamol in both mobile and immobilized conditions (Figure S4). Moreover, protein kinase A is an important molecular target of β2‐adrenergic receptor signalling, through increasing intracellular levels of the signalling molecule cAMP [13]. Interestingly, we observed that salbutamol administration increased the expression of *PRKACA* (encoding protein kinase A) in the non‐immobilized hindleg only, whereas this was unchanged in immobilized hindleg muscles (Figure 7). Together, these observations raise the question of whether salbutamol is able to activate its downstream targets (e.g., cAMP and protein kinase A) as effectively in immobilized conditions. Indeed, recent research has revealed a stimulating role of mechanical stress in β2‐receptor induced cAMP signalling [21], a finding in line with the observations of the current study. AMPK phosphorylation was increased by β2‐adrenoreceptor signalling (similar to stimulation by adrenaline [22]) and has recently been suggested to be a major regulator of muscle tissue preservation during catabolic crises [23]. Traditionally, AMPK is viewed as having anti‐anabolic effects, as it reduces mTORC1 activity and thereby protein synthesis in healthy conditions [24, S22]. However, during periods of cellular energy shortage, upregulation of AMPK may delay muscle wasting [23, S23, S24]. This suggests that activation of AMPK contributes to the restoration of homeostasis and adequate levels of ATP production, thereby positively affecting muscle protein turnover and preservation. In the current study, phosphorylation of AMPK was significantly increased ~2.5‐fold in the non‐immobilized hindlimb by salbutamol treatment, but to a lesser degree (albeit not statistically significant) in the immobilized hindlimb (+178%; Figure 6F). These findings support the notion that particularly in catabolic conditions (as induced by caloric restriction in the current study), AMPK activation is associated with a muscle‐sparing effect. In addition, AMPK phosphorylation was expected to increase due to caloric restriction [25]. Surprisingly, caloric restriction alone did not significantly affect AMPK phosphorylation (Figure 6F), a finding that conflicts with some studies [26, S25], but aligns with others [27, S26, S27]. The discrepancies between these studies could be explained by differences in the timing of tissue collection, duration of caloric restriction or myofibre type profile of the collected skeletal muscle tissue. However, this is not yet clear and requires further research. Furthermore, independently of protein kinase A, β2‐adrenergic receptor signalling has been reported to modulate the PI3K/Akt pathway [28, S28], a key pathway in the regulation of muscle mass [7]. Salbutamol did not affect Akt‐phosphorylation in the non‐immobilized nor immobilized hindlimb (Figure 6F), which may be (partially) explained by mice being terminated in a fasted state. Alternatively, this regulatory effect may have occurred earlier on during the immobilization period, after which it may have transiently decreased as a homeostatic response. This would fit with the somewhat counterintuitive observation that the eukaryotic translation initiation pathway was not upregulated, but downregulated in skeletal muscle by salbutamol administration (Figure 6E). Indeed, previous work has shown that the changes in gene expression may lag behind the rapid changes in muscle protein turnover observed upon immobilization [29, S29], which would imply that these changes are a consequence rather than a cause of the observed muscle atrophy.

A key characteristic of muscle disuse is the rapid development of peripheral insulin resistance (i.e., impaired insulin‐stimulated muscle glucose uptake; measured as a 28% reduction in forearm glucose uptake; Figure 1D), which precedes measurable muscle atrophy. As salbutamol has been reported to increase whole body glucose disposal [9], we hypothesized that its administration would attenuate or even prevent immobilization‐induced insulin resistance. We corroborate previous work by showing a ~22% increase in whole‐body insulin sensitivity [15], which occurred following merely 2 days of treatment in our healthy young participants (Figure 1B). However, these insulin‐sensitizing effects of salbutamol did not translate to attenuation of insulin resistance in immobilized muscle tissue (Figure 1C + D). These findings suggest that either activation of the β2‐adrenoreceptor or its downstream targets is insufficient to stimulate glucose uptake in muscle in the absence of contraction. This finding is different from previous studies testing the effects of salbutamol on glucose clearance during short term rest [30, S30]. Discrepancies in findings could be explained by differences in the duration of immobilization and onset of concomitant insulin resistance. Interestingly, we observed that salbutamol treatment consistently elevated circulating insulin concentrations in both humans and mice (Figure 4B and Figure S1B), corroborating previous findings in the literature [31, S31, S32]. These observed increases appear to be, at least partially, mediated by activation of beta‐receptors on pancreatic islets [31]. As a result, salbutamol administration could lead to impairment of hepatic insulin sensitivity, potentially as a mechanism to direct glucose to skeletal muscle instead of the liver as part of the ‘fight or flight’ response. Muscle contraction increases glucose uptake, independently of insulin signalling, through a variety of mechanisms [32], including Ca^2+^‐dependent signalling [33, S33], AMPK signalling [34, S34], increased blood flow (and thereby glucose delivery) [35, S34] and mechanical stress [36, S35]. These mechanisms are of importance in the development of immobilization‐induced insulin resistance and are therefore targets of pharmacological interest. Salbutamol has been shown to affect the majority of these mechanisms [13, 37, S36]; however, data from our mouse study demonstrated for the first time that salbutamol indeed upregulated some of these mechanisms in the non‐immobilized hindlimbs, but to a lesser degree in the immobilized hindlimb. More specifically, phosphorylation of Thr172‐AMPKα (Figure 6F) and upregulation of the muscle contraction pathway (Figure 6E), which for a large part consists of proteins involved in calcium homeostasis, were regulated to a lesser extent by salbutamol upon immobilization. Both AMPK and contraction‐induced Ca^2+^ signalling may increase translocation of GLUT4 to the plasma membrane, an essential step for skeletal muscle glucose uptake [33, 34, S33, S34] and thought to be the affected site in disuse atrophy [38]. It can therefore be suggested that the lack of effect of salbutamol on glucose uptake in inactive tissue is due to its inability to regulate these mechanisms in immobilized conditions. In addition, muscle contraction increases overall blood flow (and thereby substrate delivery) into a limb but also increases the surface area between muscle tissue and blood through increased capillary recruitment [35]. The latter has been identified as an important mechanism to increase muscle glucose uptake [S35]. Interestingly, despite the increase of arterial blood flow by salbutamol (Figure S3; [13]), no effects of salbutamol on forearm glucose uptake were observed. This underlines that muscle glucose uptake is a tightly regulated process, which is dependent on an interplay between arterial substrate delivery and uptake at the muscle cell level. Indeed, immobilization may have had a negative effect on capillary recruitment, which perhaps is not targeted by salbutamol administration, rendering the drug ineffective during immobilization. Furthermore, mechanical stress in skeletal muscle tissue is another mechanism that can promote glucose uptake [36, 39, S36] but does not occur during muscle disuse. Several structural proteins (e.g., dystrophin) can detect mechanical stress [39]. Rac1 is a signalling G protein and a mediator for the downstream effects of mechanical stress, including GLUT4 translocation [36, 39, S36]. In our mouse model, *Rac1* was downregulated in the immobilized hindlimb compared to the non‐immobilized hindlimb (log2FC = −0.26, *p* < 0.001), and its expression was not significantly affected by salbutamol administration in the immobilized hindlimb. This could also be an explanation for the ineffectiveness of salbutamol treatment in immobilized limbs to upregulate muscle glucose uptake.

In addition to the stimulation of muscle growth, β2‐receptor agonist administration has been reported to increase muscle strength, at least partly via increased sarcoplasmic reticulum loading and Ca^2+^ handling [S37]. For example, the ryanodine receptor Ca^2+^ release channel type 1 (*RyR1*) facilitates the release of Ca^2+^ from the sarcoplasmic reticulum into the cytosol and initiates muscle contraction upon stimulation by an action potential [S38, S39]. Interestingly, here we demonstrate that *RyR1* was upregulated by salbutamol in the non‐immobilized hindlimb, but not the immobilized hindlimb of the mouse model (Figure 7). Besides *RyR1*, other genes encoding proteins involved in calcium handling (e.g., *SERCA* subunits or *SLN*; Figure 7) demonstrated a similar response. Although this did not translate into statistically significant effects of salbutamol administration on grip strength, these muscle transcriptome data suggest that β‐adrenergic stimulation exerts beneficial effects on the calcium‐dependent regulatory systems of muscle contraction, albeit to a lesser degree in immobilized conditions. To the best of our knowledge, there are no human data available on salbutamol's potential to inhibit muscle strength loss during episodes of disuse. We speculate that salbutamol may have a minor protective effect on muscle function during disuse or as a prehabilitation strategy [S40], yet this remains to be tested in future work employing more prolonged disuse periods.

Our findings contrast with earlier studies reporting protective effects of β₂‐agonist clenbuterol on muscle mass during immobilization, most notably those in Suzuki et al. [S41]. In our study, β₂‐agonist salbutamol increased muscle protein synthesis (Figure 5A) but failed to preserve amino acid net balance (Figure 2A,B) or muscle mass in immobilized limbs (Figure 3D,E). Several factors may account for this discrepancy. First, pharmacological differences between clenbuterol and salbutamol are relevant. Clenbuterol is long‐acting and more potent but also comes with a high risk of (cardiovascular) side effects due to its relative aspecificity. In contrast, salbutamol has a higher specificity for skeletal muscle, making this drug more clinically relevant for muscle mass preservation, with a lower risk of systemic side effects. Second, differences in muscle group selection between studies may contribute to divergent outcomes, as different muscles show varying susceptibility to disuse‐induced atrophy and metabolic alterations [S42, S43]. Thirdly, durations of immobilization differ between studies and may be as short as 3 days. Our animal model, employing 2 weeks of immobilization, may reflect more advanced stages of muscle atrophy and anabolic resistance. In line, we currently cannot rule out a potential benefit of salbutamol during more prolonged periods of muscle disuse, for example, in various clinical settings. These differences underscore the importance of context in determining the efficacy of β₂‐agonists on muscle preservation during disuse.

The clinical relevance of this work lies in the administration of salbutamol as a therapy in physically inactive patients to ameliorate disuse‐induced muscle atrophy [7]. Crucially, our findings suggest that salbutamol administration will likely not prove an effective approach for preserving both muscle mass and metabolic health in completely disused muscles for short time periods. However, salbutamol treatment in patients with reduced physical activity (rather than complete inactivity), potentially for a longer duration, could perhaps still be effective yet this remains to be tested in future research. Therapies combining salbutamol treatment with induction of muscle contraction (e.g., resistance‐type exercise and/or neuromuscular electrical stimulation) are of specific interest [S44, S45], particularly for patients unable to perform voluntary contractions (e.g., in patients undergoing limb immobilization following injury or during mechanical ventilation). The combination of β2‐adrenoceptor agonists with resistance‐type exercise shows promising effectiveness, as demonstrated repeatedly by Caruso and colleagues in human immobilization models [S46, S47].

We conclude that 2 days of forearm immobilization leads to the rapid development of insulin resistance and negative amino acid net balance in forearm muscle tissue, indicative of early muscle protein loss and metabolic deterioration. Although salbutamol is highly potent in stimulating muscle mass maintenance and metabolic health in non‐immobilized muscle tissue, the drug is very unlikely to be effective in completely immobilized patients. Our findings suggest that its application should be combined with at least some degree of muscle contraction (e.g., neuromuscular electrical stimulation) to be effective.

## Conflicts of Interest

The authors declare no conflicts of interest.

## Supporting information


**Figure S1:** Salbutamol increased fasting insulin concentrations. (A) Fasting glucose and (B) insulin concentrations. Interaction *p* values were determined by three‐way ANOVA. Data of placebo (grey bars, *n* = 9) and salbutamol (blue bars, *n* = 11) groups before (open bars) and immediately after (hatched bars) immobilization are expressed as means ± SEM.

**Figure S2:** Arterialized plasma amino acid values before and after immobilization. Amino acid concentrations in the postabsorptive state (−30–0 min) and during a 3‐h hyperinsulinaemic–hyperaminoacidaemic–euglycaemic clamp (0–180 min). (A + B) Leucine concentrations, (C + D) phenylalanine concentrations, and (E + F) L‐[*ring*‐^2^H_5_]phenylalanine enrichments. *p < 0.05 as determined by three‐way ANOVA. Data of placebo (grey bars, *n* = 9) and salbutamol (blue bars, *n* = 11) groups before (open bars) and immediately after (hatched bars) immobilization are expressed as means ± SEM Data collected during both the postabsorptive state and the steady state of a hyperinsulinaemic–hyperaminoacidaemic–euglycaemic clamp are presented as indicated.

**Figure S3:** Salbutamol administration increased branchial arterial blood flow into immobilized forearm. Branchial arterial blood flow into the immobilized forearm in the (A) placebo and (B) salbutamol‐treated groups. *p < 0.05 as determined by three‐way ANOVA. Data of placebo (*n* = 9) and salbutamol (*n* = 11) groups before (white dots) and immediately after (black dots) immobilization, are expressed as means ± SEM Data are presented as collected during the hyperinsulinaemic–hyperaminoacidaemic–euglycemic clamp.

**Figure S4:** Gene expression of atrogenes F*bxo32* and *Trim63*. Normalized gene expression of (A) *Fbxo32* and (B) *Trim63*.

**Figure S5:** Western blot membranes. Western blot membranes of (A) Thr172‐AMPK and AMPKα, (B) Ser473‐Akt and total Akt and (C) LC3‐I/II.

**Data S1:** Supplementary references cited throughout the paper.

**Table S1:** Participants' characteristics.

**Table S2:** Dietary intake.

## Data Availability

RNA‐seq derived data can be accessed from the Gene Expression Omnibus (GEO) with accession number GSE278016. All other data are available from the corresponding author upon reasonable request.
