# Supplementary figures and images for: Anabolic Effects of Salbutamol Are Lost Upon Immobilization
Source: J Cachexia Sarcopenia Muscle. 2025 Nov 6;16(6):e70114. doi: 10.1002/jcsm.70114 (PMC12589897; doi:10.1002/jcsm.70114)

Supplementary figure 1

A

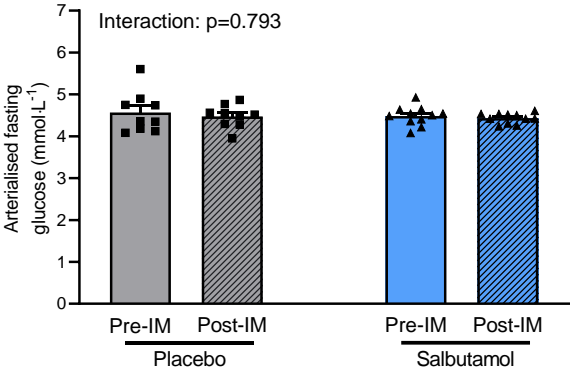

B

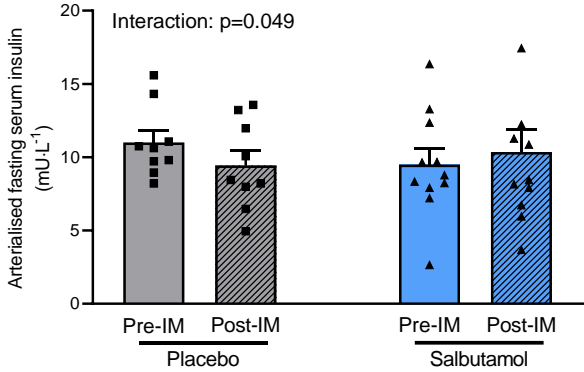

Supplement: Supplementary file 1 — Figure S1: Salbutamol increased fasting insulin concentrations. (A) Fasting glucose and (B) insulin concentrations. Interaction p values were determined by three‐way ANOVA. Data of placebo (grey bars, n = 9) and salbutamol (blue bars, n = 11) groups before (open bars) and immediately after (hatched bars) immobilization are expressed as means ± SEM. [file JCSM-16-e70114-s002.pdf]

Placebo

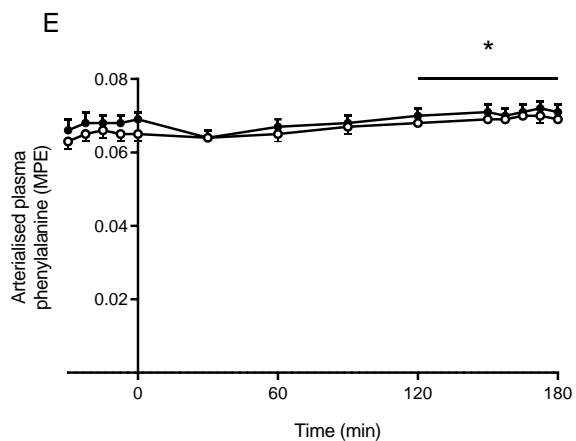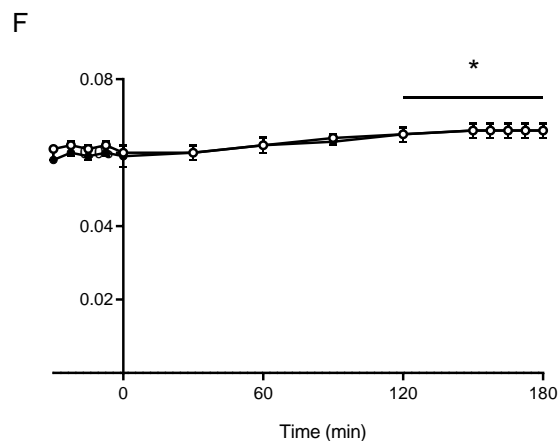

Supplement: Supplementary file 2 — Figure S2: Arterialized plasma amino acid values before and after immobilization. Amino acid concentrations in the postabsorptive state (−30–0 min) and during a 3‐h hyperinsulinaemic–hyperaminoacidaemic–euglycaemic clamp (0–180 min). (A + B) Leucine concentrations, (C + D) phenylalanine concentrations, and (E + F) L‐[ring‐2H5]phenylalanine enrichments. *p < 0.05 as determined by three‐way ANOVA. Data of placebo (grey bars, n = 9) and salbutamol (blue bars, n = 11) groups before (open bars) and immediately after (hatched bars) immobilization are expressed as means ± SEM Data collected during both the postabsorptive state and the steady state of a hyperinsulinaemic–hyperaminoacidaemic–euglycaemic clamp are presented as indicated. [file JCSM-16-e70114-s001.pdf]

Supplementary figure 3

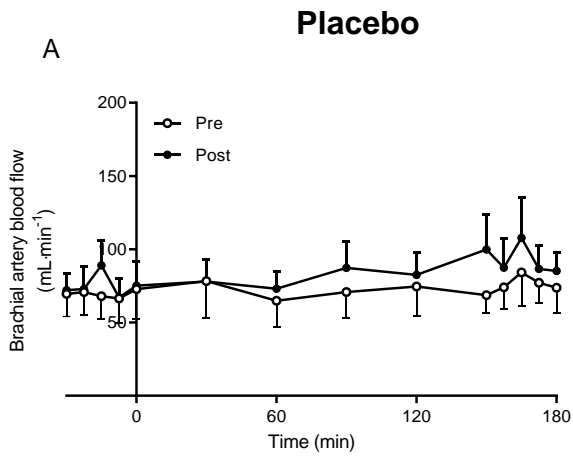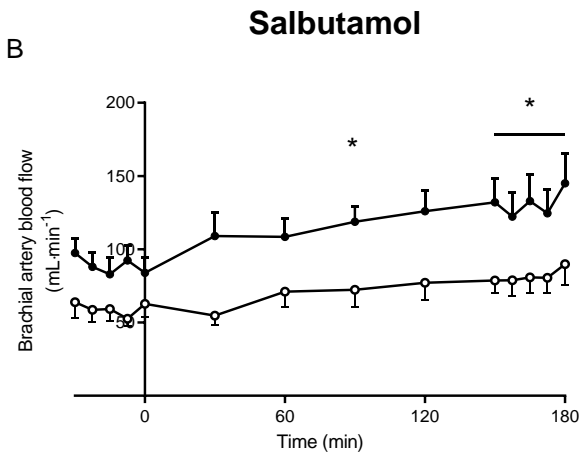

Supplement: Supplementary file 3 — Figure S3: Salbutamol administration increased branchial arterial blood flow into immobilized forearm. Branchial arterial blood flow into the immobilized forearm in the (A) placebo and (B) salbutamol‐treated groups. *p < 0.05 as determined by three‐way ANOVA. Data of placebo (n = 9) and salbutamol (n = 11) groups before (white dots) and immediately after (black dots) immobilization, are expressed as means ± SEM Data are presented as collected during the hyperinsulinaemic–hyperaminoacidaemic–euglycemic clamp. [file JCSM-16-e70114-s007.pdf]

Supplementary figure 4

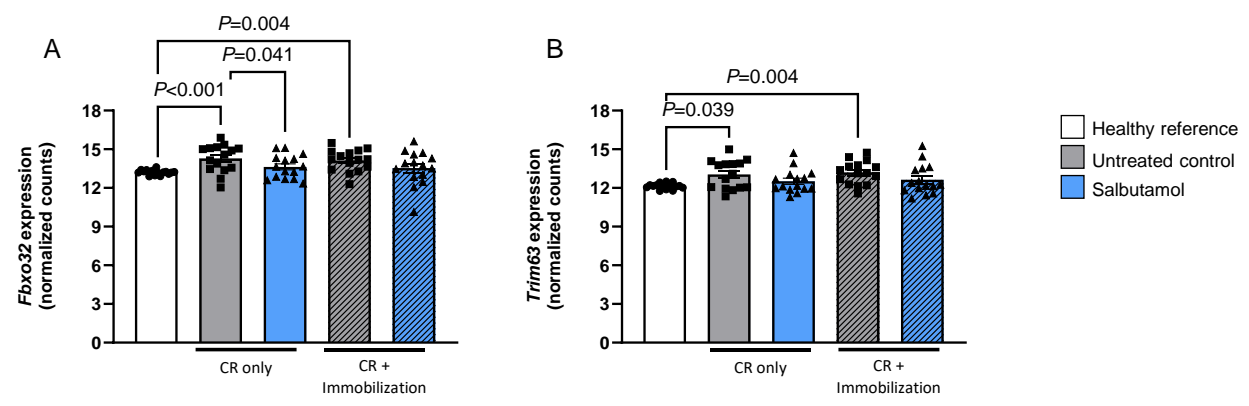

Supplement: Supplementary file 4 — Figure S4: Gene expression of atrogenes Fbxo32 and Trim63. Normalized gene expression of (A) Fbxo32 and (B) Trim63. [file JCSM-16-e70114-s005.pdf]
