# Supplementary material for: Anabolic Effects of Salbutamol Are Lost Upon Immobilization
Source: J Cachexia Sarcopenia Muscle. 2025 Nov 6;16(6):e70114. doi: 10.1002/jcsm.70114 (PMC12589897; doi:10.1002/jcsm.70114)

Supplementary figure 5

Numbers on membranes	Left leg (mobile)	Right leg (immobile)
Healthy reference	1-15	61-75
Untreated control	16-30	76-90
Salbutamol	31-45	91-105
Another study	46-60	106-120

A

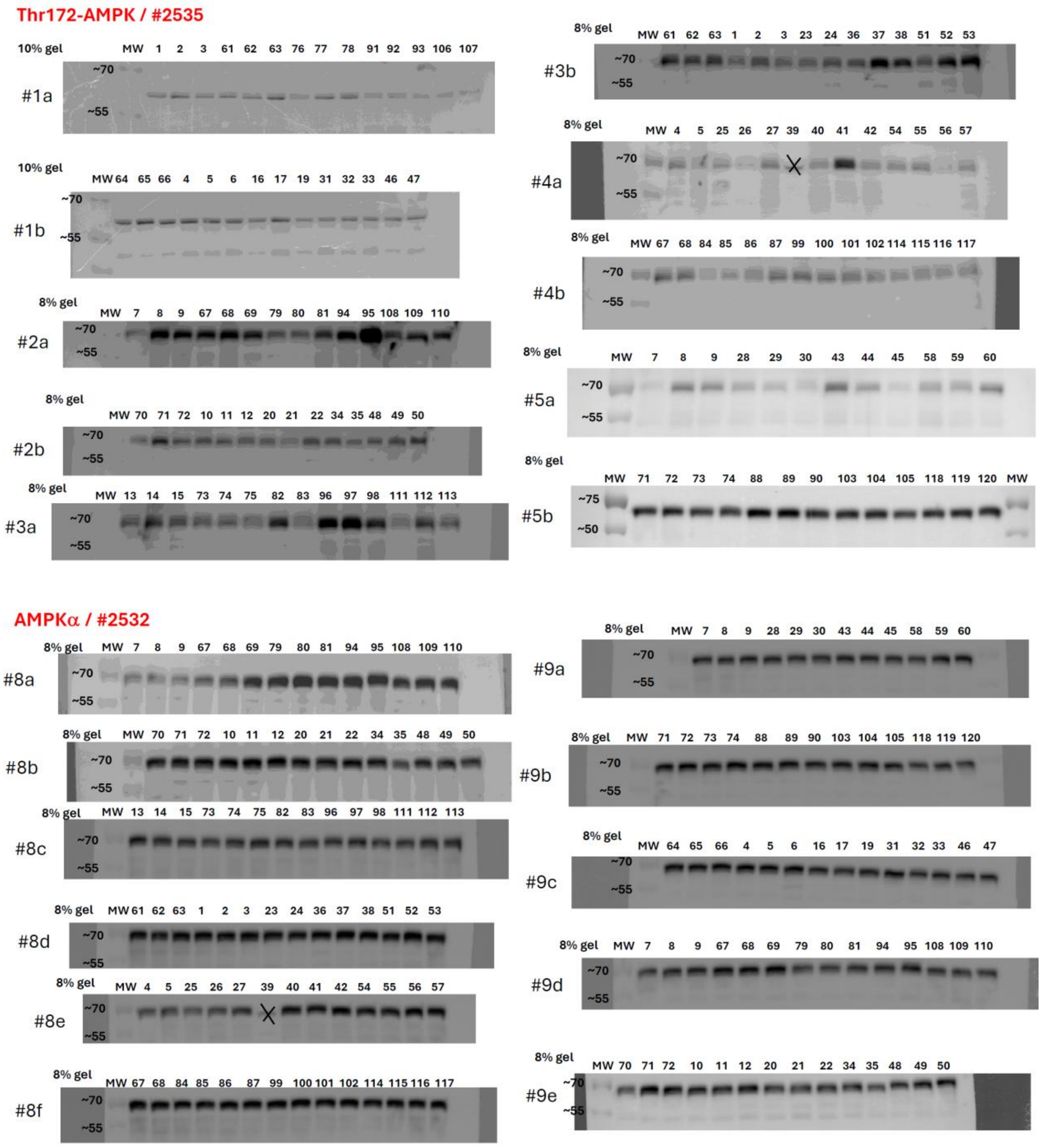

Supplementary figure 5

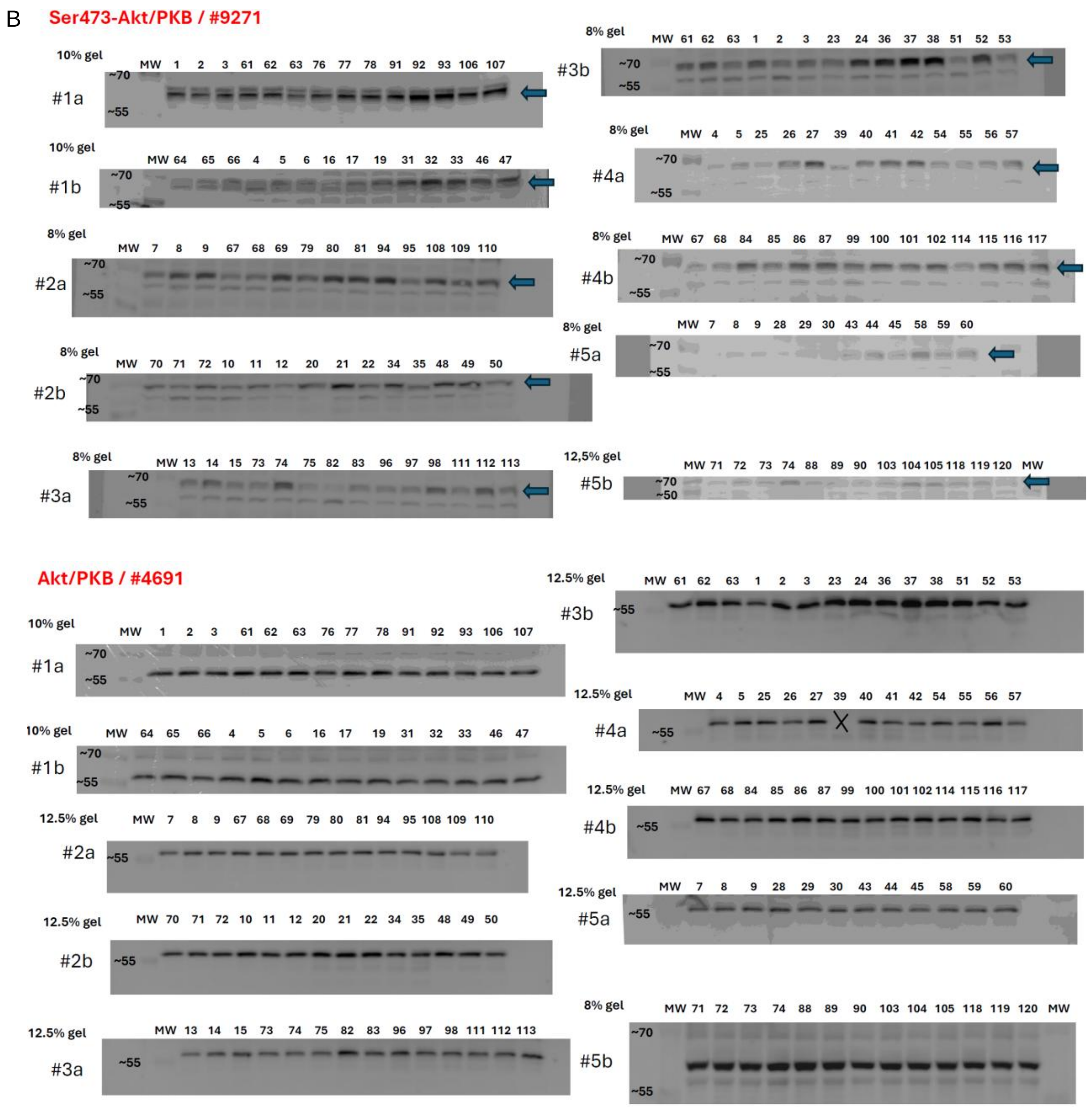

Supplementary figure 5

C

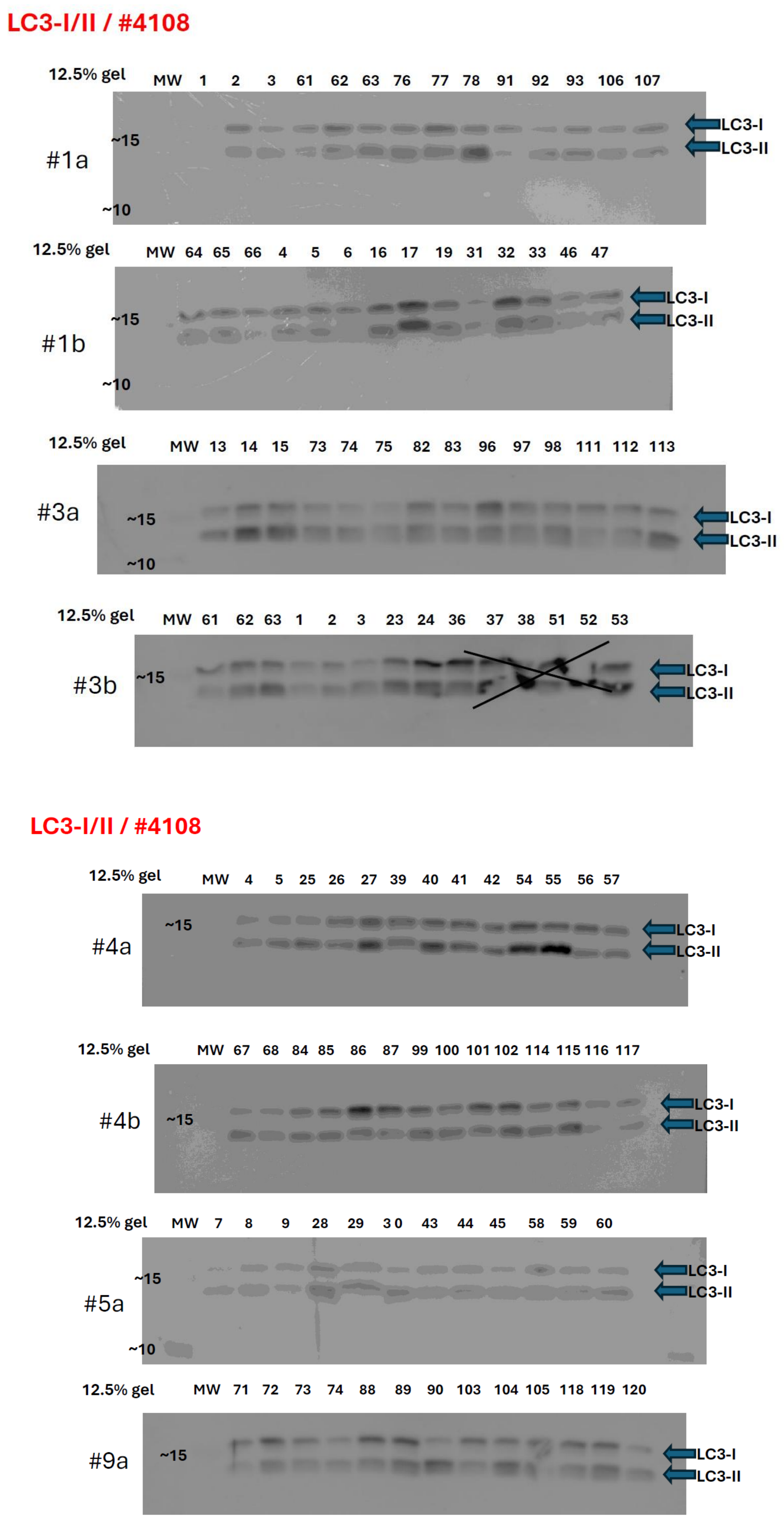

Supplement: Supplementary file 5 — Figure S5: Western blot membranes. Western blot membranes of (A) Thr172‐AMPK and AMPKα, (B) Ser473‐Akt and total Akt and (C) LC3‐I/II. [file JCSM-16-e70114-s008.pdf]
