# Supplementary material for: Anabolic Effects of Salbutamol Are Lost Upon Immobilization
Source: J Cachexia Sarcopenia Muscle. 2025 Nov 6;16(6):e70114. doi: 10.1002/jcsm.70114 (PMC12589897; doi:10.1002/jcsm.70114)
Supplement: Supplementary file 6 — Data S1: Supplementary references cited throughout the paper. [file JCSM-16-e70114-s006.docx]

**Supplemental references**

S1 Alibegovic AC, Højbjerre L, Sonne MP, Van Hall G, Stallknecht B, Dela F, et al. Impact of 9 days of bed rest on hepatic and peripheral insulin action, insulin secretion, and whole-body lipolysis in healthy young male offspring of patients with type 2 diabetes. Diabetes 2009;58:2749–56. <https://doi.org/10.2337/db09-0369>.

S2 Stuart CA, Shangraw RE, Prince MJ, Peters EJ, Wolfe RR. Bed-rest-induced insulin resistance occurs primarily in muscle. Metabolism 1988;37:802–6. <https://doi.org/10.1016/0026-0495(88)90018-2>.

S3 Glover EI, Phillips SM, Oates BR, Tang JE, Tarnopolsky MA, Selby A, et al. Immobilization induces anabolic resistance in human myofibrillar protein synthesis with low and high dose amino acid infusion. J Physiol 2008;586:6049–61. <https://doi.org/10.1113/jphysiol.2008.160333>.

S4 Joassard OR, Durieux AC, Freyssenet DG. β2-Adrenergic agonists and the treatment of skeletal muscle wasting disorders. Int J Biochem Cell Biol 2013;45:2309–21. <https://doi.org/10.1016/j.biocel.2013.06.025>.

S5 Claeys MC, Mulvaney DR, McCarthy FD, Gore MT, Marple DN, Sartin JL. Skeletal muscle protein synthesis and growth hormone secretion in young lambs treated with clenbuterol. J Anim Sci 1989;67:2245–54. <https://doi.org/10.2527/jas1989.6792245x>.

S6 Ghrayyib AS, Lilienthal JL. Measurement of blood flow and volume in the forearm of man; with notes on the theory of indicator-dilution and on production of turbulence, hemolysis, and vasodilatation by intra-vascular injection. J Clin Invest 1953;33:482–504.

S7 Gallen IW, Macdonald IA. Effect of two methods of hand heating on body temperature, forearm blood flow, and deep venous oxygen saturation. Am J Physiol - Endocrinol Metab 1990;259:E639–43.

S8 Mancini GBJ, Yeoh E, Abbott D, Chan S. Validation of an automated method for assessing brachial artery endothelial dysfunction. Can J Cardiol 2002;18:259–62.

S9 Zierler KL. Theory of the Use of Arteriovenous Concentration Differences for Measuring Metabolism in Steady and Non-Steady States*. J Clin Invest 1961;40:2111–25. <https://doi.org/10.1172/jci104437>.

S10 Henry C. Basal metabolic rate studies in humans: measurement and development of new equations. Public Health Nutr 2005;8:1133–52. <https://doi.org/10.1079/phn2005801>.

S11 Craig CL, Marshall AL, Sjöström M, Bauman AE, Booth ML, Ainsworth BE, et al. International physical activity questionnaire: 12-Country reliability and validity. Med Sci Sports Exerc 2003;35:1381–95. <https://doi.org/10.1249/01.MSS.0000078924.61453.FB>.

S12 van den Hoek AM, de Jong JCBC, Worms N, van Nieuwkoop A, Voskuilen M, Menke AL, et al. Diet and exercise reduce pre-existing NASH and fibrosis and have additional beneficial effects on the vasculature, adipose tissue and skeletal muscle via organ-crosstalk. Metabolism 2021;124:154873. <https://doi.org/10.1016/j.metabol.2021.154873>.

S13 Miller BF, Reid JJ, Price JC, Lin HJL, Atherton PJ, Smith K. CORP: The use of deuterated water for the measurement of protein synthesis. J Appl Physiol 2020;128:1163–76. <https://doi.org/10.1152/japplphysiol.00855.2019>.

S14 Gasier HG, Fluckey JD, Previs SF. The application of 2H2O to measure skeletal muscle protein synthesis. Nutr Metab 2010;7:1–8. <https://doi.org/10.1186/1743-7075-7-31>.

S15 Klein M, Vaes WHJ, Fabriek B, Sandman H, Mous DJW, Gottdang A. The 1 MV multi-element AMS system for biomedical applications at the Netherlands Organization for Applied Scientific Research (TNO). Nucl Instruments Methods Phys Res Sect B Beam Interact with Mater Atoms 2013;294:14–7. <https://doi.org/10.1016/j.nimb.2012.06.024>.

S16 Van Duijn E, Sandman H, Grossouw D, Mocking JAJ, Coulier L, Vaes WHJ. Automated combustion accelerator mass spectrometry for the analysis of biomedical samples in the low attomole range. Anal Chem 2014;86:7635–41. <https://doi.org/10.1021/ac5015035>.

S17 Thomas A, Belaidi E, Moulin S, Horman S, Van Der Zon GC, Viollet B, et al. Chronic intermittent hypoxia impairs insulin sensitivity but improves whole-body glucose tolerance by activating skeletal muscle AMPK. Diabetes 2017;66:2942–51. <https://doi.org/10.2337/db17-0186>.

S18 Milacic M, Beavers D, Conley P, Gong C, Gillespie M, Griss J, et al. The Reactome Pathway Knowledgebase 2024. Nucleic Acids Res 2024;52:D672–8. <https://doi.org/10.1093/nar/gkad1025>.

S19 Pan SJ, Hancock J, Ding Z, Fogt D, Lee M, Ivy JL. Effects of clenbuterol on insulin resistance in conscious obese Zucker rats. Am J Physiol - Endocrinol Metab 2001;280:554–61. <https://doi.org/10.1152/ajpendo.2001.280.4.e554>.

S20 Castle A, Yaspelkis BB, Kuo CH, Ivy JL. Attenuation of insulin resistance by chronic β2-adrenergic agonist treatment possible muscle specific contributions. Life Sci 2001;69:599–611. <https://doi.org/10.1016/S0024-3205(01)01149-3>.

S21 Brook MS, Stokes T, Gorissen SHM, Bass JJ, McGlory C, Cegielski J, et al. Declines in muscle protein synthesis account for short-term muscle disuse atrophy in humans in the absence of increased muscle protein breakdown. J Cachexia Sarcopenia Muscle 2022;13:2005–16. <https://doi.org/10.1002/jcsm.13005>.

S22 Lantier L, Mounier R, Leclerc J, Pende M, Foretz M, Viollet B. Coordinated maintenance of muscle cell size control by AMP-activated protein kinase. FASEB J 2010;24:3555–61. <https://doi.org/10.1096/fj.10-155994>.

S23 Hall DT, Griss T, Ma JF, Sanchez BJ, Sadek J, Tremblay AMK, et al. The AMPK agonist 5‐aminoimidazole‐4‐carboxamide ribonucleotide (AICAR), but not metformin, prevents inflammation‐associated cachectic muscle wasting. EMBO Mol Med 2018;10:1–23. <https://doi.org/10.15252/emmm.201708307>.

S24 Pigna E, Berardi E, Aulino P, Rizzuto E, Zampieri S, Carraro U, et al. Aerobic Exercise and Pharmacological Treatments Counteract Cachexia by Modulating Autophagy in Colon Cancer. Sci Rep 2016;6:1–14. <https://doi.org/10.1038/srep26991>.

S25 Palacios OM, Carmona JJ, Michan S, Chen KY, Manabe Y, Ward JL, et al. Diet and exercise signals regulate SIRT3 and activate AMPK and PGC-1alpha in skeletal muscle. Aging (Albany NY) 2009;1:771–83. <https://doi.org/10.18632/aging.100075>.

S26 Wijngaarden MA, van der Zon GC, van Dijk KW, Pijl H, Guigas B. Effects of prolonged fasting on AMPK signaling, gene expression, and mitochondrial respiratory chain content in skeletal muscle from lean and obese individuals. Am J Physiol - Endocrinol Metab 2013;304:E1012–21.

S27 Gonzalez AA, Kumar R, Mulligan JD, Davis AJ, Weindruch R, Saupe KW. Metabolic adaptations to fasting and chronic caloric restriction in heart, muscle, and liver do not include changes in AMPK activity. Am J Physiol - Endocrinol Metab 2004;287:1032–7. <https://doi.org/10.1152/ajpendo.00172.2004>.

S28 Schmidt P, Holsboer F, Spengler D. β2-adrenergic receptors potentiate glucocorticoid receptor transactivation via G protein βγ-subunits and the phosphoinositide 3-kinase pathway. Mol Endocrinol 2001;15:553–64. <https://doi.org/10.1210/me.15.4.553>.

S29 Kilroe SP, Fulford J, Holwerda AM, Jackman SR, Lee BP, Gijsen AP, et al. Short-term muscle disuse induces a rapid and sustained decline in daily myofibrillar protein synthesis rates. Am J Physiol - Endocrinol Metab 2020;318:E117–30. <https://doi.org/10.1152/ajpendo.00360.2019>.

S30 Onslev J, Thomassen M, Wojtaszewski J, Bangsbo J, Hostrup M. Salbutamol Increases Leg Glucose Uptake and Metabolic Rate but not Muscle Glycogen Resynthesis in Recovery from Exercise. J Clin Endocrinol Metab 2022;107:E1193–203. <https://doi.org/10.1210/clinem/dgab752>.

S31 Lundquist I, Ericson LE. Cell and Tissue Research fl-Adrenergic Insulin Release and Adrenergic Innervation of Mouse Pancreatic Islets. Cell Tiss Res 1978;193:73–85.

S32 Massara F, Fassio V, Camanni F, Martina V, Molinatti G. Some metabolic and hormonal effects of salbutamol in man. Acta Diabetol Lat 1976;13:146–53. <https://doi.org/10.1007/BF02581259>.

S33 Park DR, Park KH, Kim BJ, Yoon CS, Kim UH. Exercise ameliorates insulin resistance via Ca2+ signals distinct from those of insulin for GLUT4 translocation in skeletal muscles. Diabetes 2015;64:1224–34. <https://doi.org/10.2337/db14-0939>.

S34 Friedrichsen M, Mortensen B, Pehmøller C, Birk JB, Wojtaszewski JFP. Exercise-induced AMPK activity in skeletal muscle: Role in glucose uptake and insulin sensitivity. Mol Cell Endocrinol 2013;366:204–14. https://doi.org/10.1016/j.mce.2012.06.013.

S35 Schultz TA, Lewis SB, Westbie DK, Gerich JE, Rushakoff RJ, Wallin john D. Glucose delivery - A clarification of its role in regulating glucose uptake in rat skeletal muscle. Life Sci 1977;20:733–5.

S36 Sylow L, Jensen TE, Kleinert M, Mouatt JR, Maarbjerg SJ, Jeppesen J, et al. Rac1 is a novel regulator of contraction-stimulated glucose uptake in skeletal muscle. Diabetes 2013;62:1139–51. https://doi.org/10.2337/db12-0491.

S37 Cairns SP, Borrani F. β-Adrenergic modulation of skeletal muscle contraction: Key role of excitation-contraction coupling. J Physiol 2015;593:4713–27. <https://doi.org/10.1113/JP270909>.

S38 Schneider MF, Chandler WK. Voltage Dependent Charge Movement in Skeletal Muscle: a Possible Step in Excitation-Contraction Coupling. Nature 1973;242:244–6.

S39 Block BA, Imagawa T, Campbell KP, Franzini-Armstrong C. Structural evidence for direct interaction between the molecular components of the transverse tubule/sarcoplasmic reticulum junction in skeletal muscle. J Cell Biol 1988;107:2587–600. <https://doi.org/10.1083/jcb.107.6.2587>.

S40 Smeuninx B, Elhassan YS, Sapey E, Rushton AB, Morgan PT, Korzepa M, et al. A single bout of prior resistance exercise attenuates muscle atrophy and declines in myofibrillar protein synthesis during bed-rest in older men. J Physiol 2023:1–19. <https://doi.org/10.1113/JP285130>.

S41 Suzuki H, Kitaura T. Attenuating effects of clenbuterol, β2-agonist, on immobilization - induced atrophy of rat hindlimb muscle fibers. J Phys Fit Sport Med 2015;4:363–7. <https://doi.org/10.7600/jpfsm.4.363>.

S42 Belavý DL, Miokovic T, Armbrecht G, Richardson CA, Rittweger J, Felsenberg D. Differential atrophy of the lower-limb musculature during prolonged bed-rest. Eur J Appl Physiol 2009;107:489–99. https://doi.org/10.1007/s00421-009-1136-0.

S43 Kilroe SP, Fulford J, Holwerda AM, Jackman SR, Lee BP, Gijsen AP, et al. Short-term muscle disuse induces a rapid and sustained decline in daily myofibrillar protein synthesis rates. Am J Physiol - Endocrinol Metab 2020;318:E117–30. https://doi.org/10.1152/ajpendo.00360.2019.

S44 Dirks ML, Hansen D, Van Assche A, Dendale P, Van Loon LJC. Neuromuscular electrical stimulation prevents muscle wasting in critically ill comatose patients. Clin Sci 2015;128:357–65. https://doi.org/10.1042/CS20140447.

S45 Dirks ML, Wall BT, Snijders T, Ottenbros CLP, Verdijk LB, Van Loon LJC. Neuromuscular electrical stimulation prevents muscle disuse atrophy during leg immobilization in humans. Acta Physiol 2014;210:628–41. https://doi.org/10.1111/apha.12200.

S46 Caruso JF, Hamill JL, Yamauchi M, Mercado DR, Cook TD, Keller CP, et al. Albuterol helps resistance exercise attenuate unloading-induced knee extensor losses. Aviat Sp Environ Med 2004;75:505–11.

S47 Caruso JF, Hamill JL, Yamauchi M, Saito K, Cook TD, Mercado DR. Temporal strength changes from resistance exercise and albuterol on unloaded muscle. J Strength Cond Res 2008;22:1156–63. https://doi.org/10.1519/JSC.0b013e31816eb46a.
