# Supplementary material for: Anabolic Effects of Salbutamol Are Lost Upon Immobilization
Source: J Cachexia Sarcopenia Muscle. 2025 Nov 6;16(6):e70114. doi: 10.1002/jcsm.70114 (PMC12589897; doi:10.1002/jcsm.70114)
Supplement: Supplementary file 7 — Table S1: Participants' characteristics. [file JCSM-16-e70114-s003.docx]

**Supplemental table 1: Participants’ characteristics**

|  | Placebo (*n*=9) | Salbutamol (*n*=11) |
| --- | --- | --- |
| Sex (M/F) | 5 / 4 | 6 / 5 |
| Age (y) | 23 ± 2 | 23 ± 1 |
| Height (cm) | 175 ± 3 | 175 ± 3 |
| Body mass (kg) | 72.9 ± 3.1 | 74.2 ± 4.1 |
| BMI (kg·m^-2^) | 23.9 ± 0.7 | 24.1 ± 1.0 |
| Body fat (% of body mass) | 25.1 ± 3.1 | 24.4 ± 3.6 |
| Lean mass (kg) | 54.8 ± 3.8 | 55.5 ± 3.4 |
| Systolic blood pressure (mm Hg) | 114 ± 4 | 111 ± 3 |
| Diastolic blood pressure (mm Hg) | 65 ± 2 | 70 ± 2 |

None of these variables were statistically different (p<0.05) between groups
