# Supplementary material for: Anabolic Effects of Salbutamol Are Lost Upon Immobilization
Source: J Cachexia Sarcopenia Muscle. 2025 Nov 6;16(6):e70114. doi: 10.1002/jcsm.70114 (PMC12589897; doi:10.1002/jcsm.70114)
Supplement: Supplementary file 8 — Table S2: Dietary intake. [file JCSM-16-e70114-s004.docx]

**Supplemental table 2: Dietary intake**

|  | Placebo (*n*=9) | | Salbutamol (*n*=11) | |
| --- | --- | --- | --- | --- |
|  | **Habitual** | **Immobilization** | **Habitual** | **Immobilization** |
| Energy (MJ·d^-1^) | 9.4 ± 0.9 | 11.6 ± 0.6 * | 9.3 ± 0.7 | 11.1 ± 0.5 * |
| Protein (g·kg^-1^·d^-1^) | 1.19 ± 0.12 | 1.22 ± 0.02 | 1.14 ± 0.11 | 1.21 ± 0.00 |
| Protein (g·d^-1^) | 88 ± 9 | 91 ± 4 | 83 ± 8 | 90 ± 5 |
| Carbohydrates (g·d^-1^) | 224 ± 18 | 371 ± 24 * | 254 ± 30 | 345 ± 15 * |
| Fat (g·d^-1^) | 86 ± 14 | 96 ± 4 | 94 ± 9 | 95 ± 5 |
| Fibres (g·d^-1^) | 21 ± 3 | 32 ± 2 * | 22 ± 3 | 28 ± 1 * |
| Alcohol (g·d^-1^) | 18 ± 12 | 0 ± 0 | 4 ± 2 $ | 0 ± 0 |
| Protein (En%) | 17 ± 1 | 13 ± 0 * | 15 ± 1 | 14 ± 0 * |
| Carbohydrate (En%) | 42 ± 2 | 53 ± 1 * | 44 ± 3 | 52 ± 1 * |
| Fat (En%) | 35 ± 3 | 31 ± 1 * | 38 ± 2 | 32 ± 0 * |
| Fibres (En%) | 1.9 ± 0.2 | 2.3 ± 0.1 * | 1.9 ± 0.1 | 2.1 ± 0.1 * |
| Alcohol (En%) | 5 ± 3 | 0 ± 0 * | 2 ± 1 | 0 ± 0 * |

En%, energy percentage; MJ, MegaJoule. * Significantly different from habitual intake value (*P*<0.05). $ Significantly different from corresponding habitual dietary intake value in placebo group (*P*<0.05).
